# Rat brain phospholipid remodeling correlates with plasma lipid clearance and late functional vulnerability after ischemic stroke

**DOI:** 10.1016/j.isci.2026.117434

**Published:** 2026-09-05

**Authors:** Iván Daniel Salomón-Cruz, Johanna Gutiérrez-Vargas, Gloria Patricia Cardona-Gómez

**Affiliations:** 1 Cellular and Molecular Neurobiology Area, Group of Neuroscience of Antioquia, Faculty of Medicine, University of Antioquia, Medellín, Colombia

**Keywords:** phospholipids profile, membrane remodeling, lipid clearance, lipoproteins, post-stroke, post-stroke cognitive impairment, PSCI

## Abstract

Post-stroke neurological decline is associated with persistent vascular and metabolic dysfunction, including long-term alterations in lipid homeostasis. We performed targeted lipidomic analyses in the hippocampus and plasma from rats subjected to transient middle cerebral artery occlusion at 6 h, 24 h, 7 days, 15 days, 1 month, and 4 months after stroke. Lipid profiles were integrated with behavioral, histological, immunofluorescence, western blot, flow cytometric, and bioinformatic analyses. At 4 months, an increased plasma lysophosphatidylcholine/phosphatidylcholine ratio was associated with late neurological deterioration and cognitive impairment. This change coincided with hippocampal lipid remodeling, region-specific astrogliosis, changes in PLA2, LPCAT1, MBOAT1, and SREBF2, and altered circulating BODIPY^+^ lipophilic particles associated with apolipoproteins. These findings support coordinated central and peripheral lipid remodeling during chronic post-ischemic progression with potential relevance for peripheral biomarker development.

## Highlights

• Plasma phospholipid remodeling underlies the late neurological vulnerability after stroke
• Land’s cycle enzymes and lipid storage implied at the post-ischemic brain susceptibility
• Brain-to-blood “lipid clearance” following late post-ischemic stroke
• LPC/PC as potential prognostic biomarker of neurovascular dysfunction and cognitive impairment

## Graphical abstract

**Figure:**
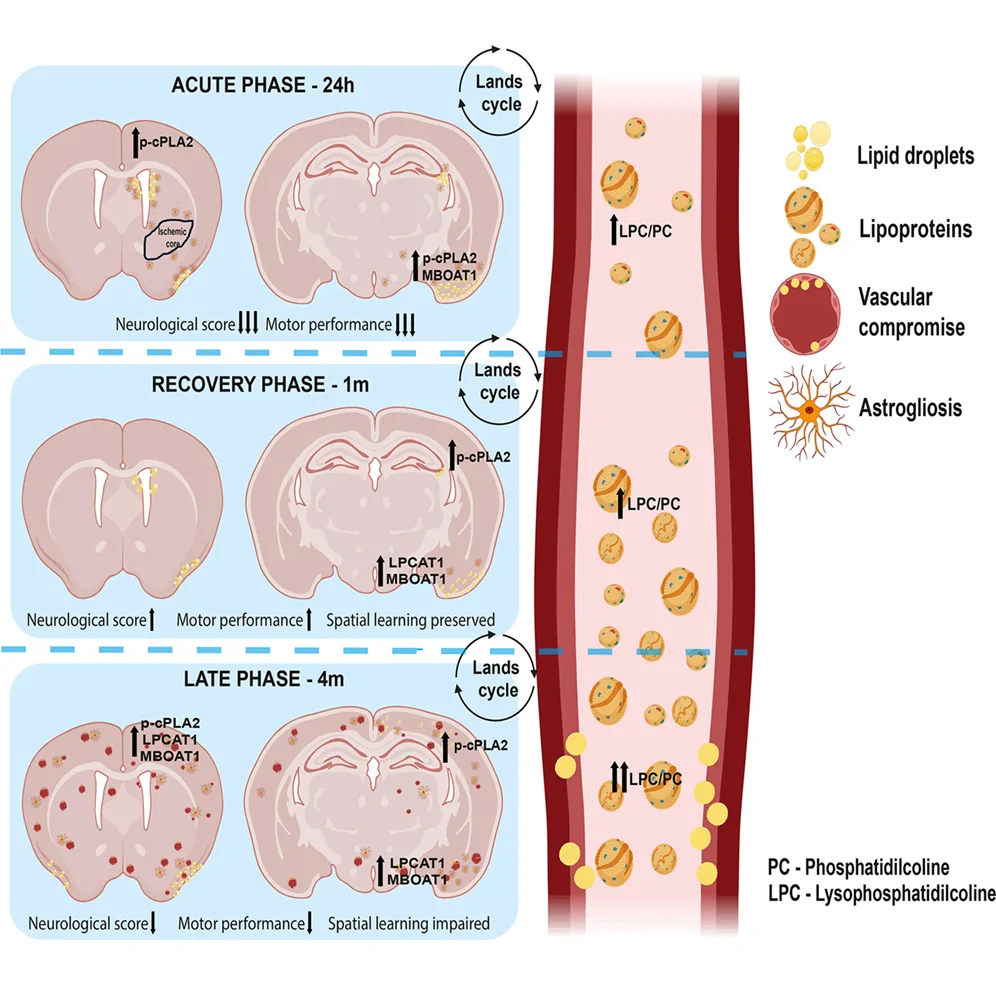

## Teaser

Health sciences; Medicine; Medical specialty; Internal medicine; Neurology

## Introduction

Ischemic stroke remains one of the leading causes of disability and mortality worldwide, affecting millions of individuals each year. Although therapeutic interventions have advanced in mitigating the acute consequences of stroke, long-term complications, particularly cognitive decline, remain a major clinical challenge. It is estimated that 20%–30% of stroke survivors develop cognitive impairments in the years following the event, with a significant proportion progressing to vascular dementia, Alzheimer’s disease, or mixed pathologies.^1^ Post-stroke cognitive impairment (PSCI) is especially prevalent within the first year after stroke and can range from mild deficits to severe deterioration. While some individuals may experience partial recovery early on, up to one-third ultimately develop dementia within 5 years.^2^ This cognitive trajectory is thought to result from a combination of acute vascular injury and pre-existing microvascular and neurodegenerative changes, which are often exacerbated by metabolic comorbidities, such as dyslipidemia, obesity, and metabolic syndrome.^1^^,^^2^ In this context, the identification of peripheral biomarkers capable of predicting delayed PSCI is a critical unmet need. Plasma biomarkers offer a promising solution, providing a minimally invasive, cost-effective, and scalable approach for detecting early systemic and brain-derived pathophysiological alterations and guiding timely therapeutic interventions. Unlike cerebrospinal fluid (CSF) or tissue-based sampling, plasma enables longitudinal assessments, making it particularly well suited for monitoring dynamic processes that occur in the aftermath of acute neurological insults such as stroke.^3^^,^^4^

In ischemic brain injury, several plasma biomarkers have been identified as sensitive indicators of acute neuronal and glial damage, such as glial fibrillary acidic protein (GFAP) and neurofilament light chain (NfL).^5^^,^^6^ However, reliable markers for the subacute and chronic phases following stroke are still lacking. This gap is especially relevant given the increasing recognition that stroke can initiate progressive cerebral alterations associated with long-term cognitive decline. Among the emerging candidates to fill this gap, plasma lipid profiles, particularly phospholipids, have attracted increasing interest.^7^ Phospholipids are essential structural components of cellular membranes, actively participate in intracellular signaling and vesicle trafficking, and are key constituents of circulating lipoprotein particles.^8^ Disruptions in phospholipid homeostasis have been implicated in several pathophysiological processes, including inflammation, endothelial dysfunction, protein aggregation, oxidative stress, and neuronal death. These roles position phospholipids as highly relevant candidates for developing biomarkers of brain injury and neurodegeneration.^9^ Following an ischemic stroke, membrane rupture, oxidative stress, and activation of phospholipolytic enzymes alter membrane lipid composition and lipid signaling.^10^ These changes can be temporally monitored in rodent models of transient middle cerebral artery occlusion (tMCAO), which reliably replicate cerebral ischemia-reperfusion,^11^ without the confounding effects of human comorbidities.

Therefore, this study adopts a time-resolved approach to investigate lipidomic alterations following ischemic stroke, with the goal of identifying translationally relevant plasma biomarkers linked to brain dysfunction. By combining mass spectrometry-based phospholipid profiling in both plasma and hippocampal tissue from the same animals, this model enables direct assessment of the relationship between central and peripheral lipid changes. Bioinformatic pathway analysis was used to identify key enzymes and metabolic routes, while biochemical and histological analyses further supported the relevance of these findings. The use of a controlled experimental model allows for in-depth neurobiological validation of candidate mechanisms underlying post-stroke lipid remodeling, bridging the gap between preclinical observations and biomarker discovery with potential applications in human disease.

## Results

### Late neurological and cognitive impairment is associated with progressive brain alterations after tMCAO

**Figure 1:**
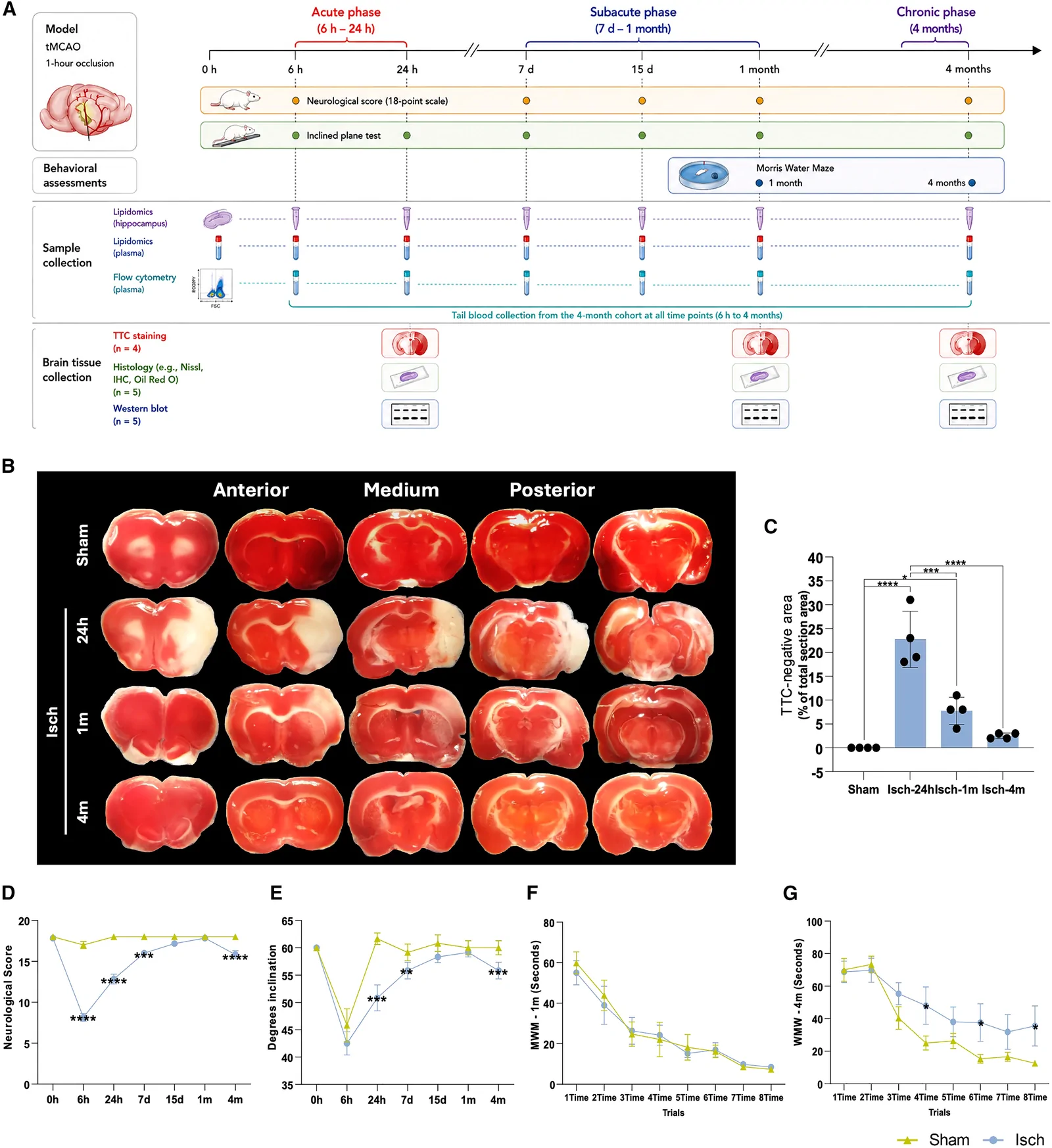
Longitudinal design: Assessment of initial tissue damage and its cognitive and motor repercussions over time in the tMCAO model (A) Longitudinal experimental design. Neurological score and inclined plane performance were assessed at 6 h, 24 h, 7 days, 15 days, 1 month, and 4 months post-ischemia. Hippocampal tissue and plasma were collected at all time points for phospholipidomic analyses. Brain tissue was collected at 24 h, 1 month, and 4 months for 2,3,5-triphenyltetrazolium chloride (TTC) staining, histological assessment, and biochemical analyses. Tail blood samples from the 4-month cohort were collected longitudinally for flow cytometry analyses. (B) Representative TTC staining from coronal sections of ischemic animals at 24 h, 1 month, and 4 months post-ischemia, with representative sham control tissue shown at 24 h. Serial sections are shown from anterior to posterior anatomical levels. (C) Quantification of corrected infarct volume (% of contralateral hemisphere volume). (D) Longitudinal neurological score assessed using an 18-point scale. (E) Longitudinal inclined plane test assessing motor/postural performance. (F and G) Morris water maze acquisition performance at 1 month (F) and 4 months (G) post-ischemia. For neurological score and inclined plane analyses, *n* = 6 animals per group and time point. For Morris water maze analyses, *n* = 5 animals per group at both 1 and 4 months. For hippocampal/plasma lipidomics analyses, *n* = 5 animals per group and time point unless otherwise indicated. TTC analysis was performed at 24 h, 1 month, and 4 months post-ischemia, with *n* = 4 animals per time point; sham control tissue was included at 24 h. Histological and western blot analyses were performed at 24 h, 1 month, and 4 months post-ischemia with *n* = 3 animals per group and time point. Corrected infarct volume was analyzed by one-way ANOVA followed by Tukey’s multiple comparisons test. Neurological score and Morris water maze acquisition at 1 month were analyzed by ordinary two-way ANOVA, whereas inclined plane performance and Morris water maze acquisition at 4 months were analyzed by two-way repeated-measures ANOVA; Šídák’s multiple comparisons test was used for sham versus ischemic comparisons within each time point or trial. Asterisks indicate corrected post hoc comparisons between sham and ischemic animals within the same time point or trial. Quantitative data are presented as mean ± SD. ∗*p* < 0.05, ∗∗*p* < 0.01, ∗∗∗*p* < 0.001, ∗∗∗∗*p* < 0.0001.

To characterize the timeline of post-ischemic injury, we used a longitudinal tMCAO design including acute, subacute, and chronic stages after reperfusion. Neurological score and inclined plane performance were assessed at 6 h, 24 h, 7 days, 15 days, 1 month, and 4 months post-ischemia, whereas spatial learning was evaluated using the Morris water maze (MWM) at 1 and 4 months. In parallel, hippocampal tissue and plasma were collected at all time points for phospholipidomic analyses, while brain tissue was collected at 24 h, 1 month, and 4 months for 2,3,5-triphenyltetrazolium chloride (TTC) staining, histological assessment, and biochemical analyses (Figure 1A).

tMCAO induced a well-defined primary ischemic lesion followed by delayed neurological deterioration. Infarct volume was evaluated by TTC staining across serial coronal sections at 24 h, 1 month, and 4 months post-ischemia (Figure 1B). At 24 h post-ischemia, TTC staining revealed a well-defined ischemic area in the ipsilateral hemisphere, primarily involving cortical and striatal territories, including the caudate-putamen, with a significantly increased TTC-negative area compared with sham animals (Figures 1B and 1C). At 1 month post-ischemia, the TTC-negative area was significantly reduced compared with 24 h but remained detectable, mainly in cortical regions. By 4 months, the TTC-negative area was minimal and no longer significantly different from sham animals (Figures 1B and 1C), supporting a temporal reduction of the primary TTC-defined lesion.^12^

These anatomical changes paralleled functional outcomes. Neurological function assessed using a composite 18-point neurological score adapted from the battery originally described by García et al.,^13^ declined sharply at 6 h post-ischemia compared to sham animals and progressively recovered by 15 days, remaining stable at 1 month (Figure 1D). Sham-operated animals exhibited a mild reduction in neurological and motor performance restricted to the 6-h time point, with scores slightly below the maximum value and a more pronounced decrease in the inclined plane test, consistent with transient post-surgical effects. Notably, a significant late decline in neurological performance was detected at 4 months post-ischemia (Figure 1D), a pattern that was also observed in the inclined plane test, indicating late deterioration in motor coordination and strength (Figure 1E). Spatial learning acquisition in the MWM was preserved at 1 month post-ischemia (Figure 1F). At 4 months, ischemic animals showed impaired acquisition performance, with significantly increased escape latency compared with sham animals at trials 4, 6, and 8 (Figure 1G).

### Dynamic phospholipid remodeling in the hippocampus during stroke progression

**Figure 2:**
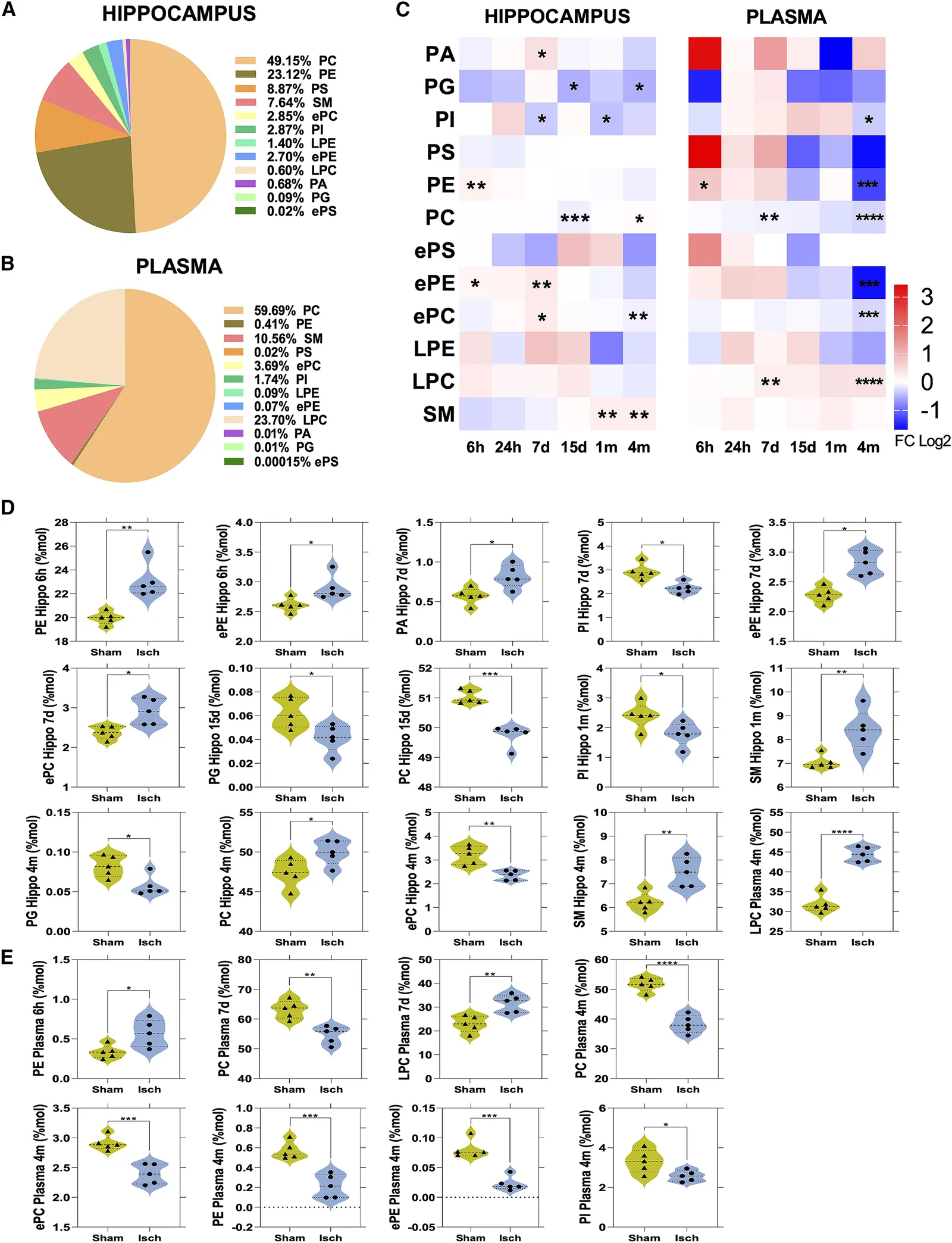
Phospholipid class alterations post-tMCAO (A and B) Average phospholipid class distribution in sham animals used as reference lipid composition for the hippocampus (A) and plasma (B), expressed as molar percentage (mol %). (C) Heatmap representation of longitudinal post-tMCAO changes in hippocampal and plasma phospholipid classes, expressed as log_2_ fold-change relative to time-matched sham controls. (D and E) Violin plots showing the distribution of significantly altered phospholipid classes in the hippocampus (D) and plasma (E) across post-tMCAO time points. For heatmap-based class comparisons in (C), statistical analysis was performed using two-way ANOVA followed by Šídák’s multiple comparisons test, comparing sham and ischemic animals within each time point. For violin plot comparisons in (D) and (E), data were analyzed using unpaired two-tailed Student’s t-tests comparing sham and ischemic animals within each time point. Data are shown for *n* = 5 animals per group and time point. Significant changes are indicated as ∗*p* < 0.05, ∗∗*p* < 0.01, ∗∗∗*p* < 0.001, and ∗∗∗∗*p* < 0.0001. See also Figures S1 and S2; Table S1.

The hippocampus was selected for lipidomic analysis due to its recognized vulnerability to post-ischemic network dysfunction and its critical role in learning and memory processes.^14^^,^^15^ Lipidomic profiling of the hippocampus and plasma, expressed as molar percentage (mol %), revealed a time-dependent remodeling of phospholipid composition across 12 lipid classes and 299 molecular species (Table S1), allowing us to evaluate relative shifts in phospholipidome organization rather than isolated abundance changes. Sham animals were used to define the reference phospholipid composition of the hippocampus and plasma. In the hippocampus, phosphatidylcholine (PC, ∼50%) and phosphatidylethanolamine (PE, ∼23%) represented the most abundant classes, followed by phosphatidylserine (PS, ∼9%) and sphingomyelin (SM, ∼8%), whereas phosphatidylinositol (PI), ether-linked phospholipids, phosphatidic acid (PA), and lysophospholipids accounted for smaller proportions of the hippocampal lipidome (Figure 2A).

To capture the global temporal dynamics of phospholipid remodeling, log_2_ fold-change heatmaps were generated for all phospholipid classes relative to time-matched sham controls, with statistically significant alterations indicated by asterisks (Figure 2C). Complementary class-level plots depict the magnitude and dispersion of phospholipid changes for classes showing significant remodeling (Figure 2D). Heatmap analysis revealed distinct temporal phases of hippocampal phospholipid remodeling following ischemia. During the acute phase (6 h post-ischemia), class-level alterations were limited and primarily involved PE and ether-linked PE (ePE), which showed early increases. At 6 h, these changes were accompanied by species-level clustering patterns involving long-chain polyunsaturated PC and PE species, together with shorter and more saturated species that distinguished sham from ischemic samples (Figure S1A), consistent with an early redistribution of membrane phospholipid composition rather than global phospholipid loss.

At 24 h post-ischemia, hippocampal phospholipid profiles showed minimal separation between ischemic and sham animals at the class level, consistent with the heatmap pattern (Figure 2C). Nonetheless, species-level hierarchical clustering still revealed lipid species patterns that distinguished subsets of ischemic and sham samples, suggesting ongoing phospholipid remodeling not captured by global class abundance (Figure S1A).

During the subacute phase (7–15 days), hippocampal phospholipid remodeling became more pronounced and involved a broader range of phospholipid classes, as reflected by coordinated changes in PA, PC, PE, and ether-linked phospholipids in the heatmap. These alterations were supported by species-level clustering patterns involving mainly intermediate-length acyl chains, consistent with active membrane reorganization during the recovery phase. Importantly, several species from classes without significant overall changes also displayed distinct clustering patterns between sham and ischemic samples, highlighting the added resolution provided by species-level analysis beyond class-level trends (Figure S1A).

At the transition to chronic phase (1 and 4 months post-ischemia), the hippocampal heatmap revealed a persistent phospholipid remodeling signature characterized by sustained reductions in PI, phosphatidylglycerol (PG), and ether-linked PC (ePC), together with a relative increase in SM and PC (Figure 2C). These late changes were accompanied by species-level patterns enriched in long-chain polyunsaturated fatty acids, including docosahexaenoic acid (DHA)-containing species, indicating prolonged alterations in membrane structure and lipid-mediated signaling. At 4 months post-ischemia, the hippocampal phospholipidome showed continued remodeling, with class-level reductions in PA and a relative enrichment of SM, consistent with sustained alterations in membrane composition. These changes temporally overlapped with late neurological deterioration. Species-level clustering patterns associated with these class-level changes are shown in Figure S1A.

### Plasma phospholipids display a dominant and progressive shift in the LPC/PC balance following ischemia

Plasma phospholipidomic profiling in sham animals revealed that PC (∼60%) and lysophosphatidylcholine (LPC, ∼24%) were the predominant phospholipid classes, followed by SM (∼11%), ether-PC (ePC, ∼4%), PI (∼2%), and PE (∼0.4%) (Figure 2B). A significant early increase in PE levels was detected at 6 h post-ischemia (Figure 2C). From this early time point onward, plasma phospholipid remodeling was dominated by reciprocal changes in PC and LPC. LPC levels progressively increased, whereas PC levels declined, resulting in a marked temporal shift in the LPC/PC ratio. These alterations constituted the most robust and statistically significant plasma phospholipid changes across the post-ischemic period, reaching significance at 7 days and becoming more pronounced at 4 months post-ischemia (Figures 2C and E).

In addition to this dominant PC/LPC shift, the chronic post-ischemia stage was characterized by a marked reduction in several minor phospholipid classes. At 4 months, significant decreases were observed in ether-linked phospholipids (ePC and ePE), PE, and PI, indicating a broader disruption of glycerophospholipid homeostasis beyond choline-containing species. In contrast, SM levels remained comparatively stable across time. PS exhibited only a transient, non-significant increase at early time points.

Consistent with the global class-level patterns observed in the plasma heatmap (Figure 2C), species-level analysis revealed distinct clustering patterns among specific phospholipid species within affected classes. Early post-ischemic time points showed species-level patterns involving LPC- and PE-derived species and reductions in multiple PC and ePC species, while the chronic phase (4 months) displayed the greatest divergence between ischemic and control animals, with marked clustering differences across PC-, ePC-, PE-, ePE-, and PI-derived species accompanied by a sustained accumulation of LPC species (Figure S1B).

### Phospholipid signatures and remodeling pathways in the post-ischemic hippocampus and plasma

Longitudinal principal component analysis (PCA) revealed distinct phospholipid signatures underlying temporal remodeling in the hippocampus and plasma following ischemia (Figure S2). In the hippocampus, the first principal component (PC1) explained 54.1%–64.6% of the variance across time points, indicating a strong temporal structuring of lipid species associated with post-ischemic progression. Early separation (6 h) was primarily driven by ethanolamine-containing phospholipids, particularly polyunsaturated PE species, whereas later time points showed increasing contributions from ePE and selected choline-containing species. At chronic stages, hippocampal PCA loadings were dominated by polyunsaturated ePE and PC species, together with contributions from SM species, reflecting sustained membrane remodeling rather than a return to the sham lipidomic state.

In plasma, PC1 accounted for 58.6%–66.6% of the variance, but the identity of discriminant species differed markedly from those observed in the hippocampus. Throughout the time course, plasma PCA separation was driven predominantly by PC- and LPC-related species, with additional contributions from ePC, ePE, SM, and PI at specific stages. Notably, LPC species became increasingly prominent contributors during subacute and chronic phases, consistent with a progressive shift in systemic phospholipid metabolism following ischemia (Figure S2).

Importantly, the limited overlap between discriminant phospholipid species identified in hippocampal and plasma PCA reflects fundamental differences in tissue-specific phospholipid remodeling rather than a lack of association between compartments. While both tissues exhibited alterations within similar phospholipid classes, dynamic fatty acid deacylation and reacylation processes, particularly within the Lands cycle, can modify molecular species composition while preserving class-level changes. Thus, ischemia-induced phospholipid remodeling is expected to manifest as class-concordant but species-divergent signatures between brain tissue and circulation.

**Figure 3:**
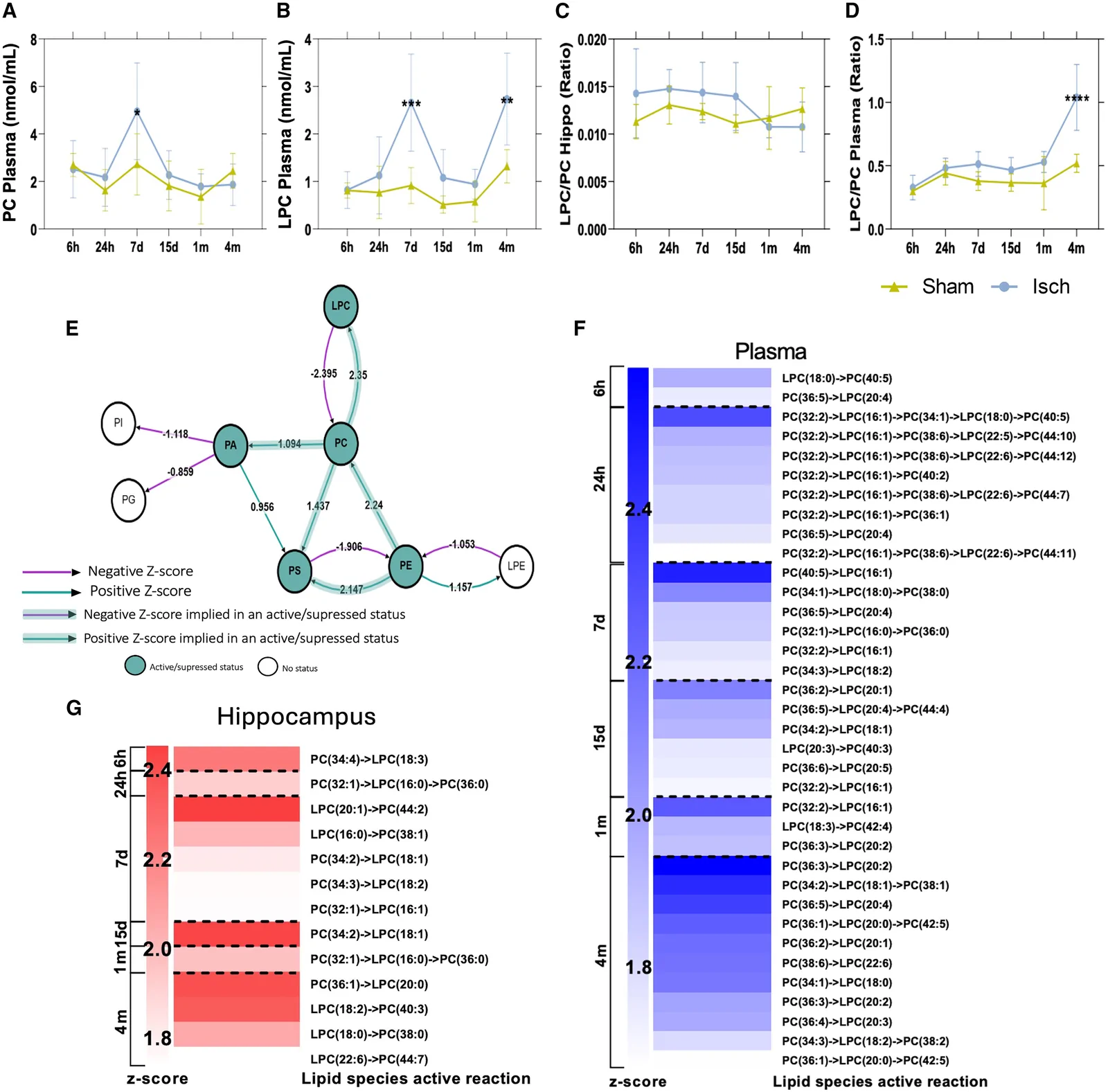
Plasma PC/LPC imbalance and BioPAN-inferred phospholipid remodeling after tMCAO (A and B) Longitudinal plasma abundance of PC (A) and LPC (B), expressed as nmol/mL. (C and D) LPC/PC ratio in the hippocampus (C) and plasma (D), calculated from molar proportions (mol %) of LPC and PC classes. (E) BioPAN-based overview of phospholipid remodeling reactions inferred from the hippocampal and plasma phospholipid species datasets. (F and G) Main PC/LPC interconversion reactions across post-ischemic time points in plasma (F) and hippocampus (G) generated using the BioPAN tool. These graphs visualize temporal changes in inferred phospholipid reactions relative to time-matched sham controls. Data are presented as *Z* scores, reflecting the standardized magnitude and direction of each inferred reaction. For plasma PC and LPC abundance and hippocampal and plasma LPC/PC ratio analyses, statistical comparisons were performed using two-way ANOVA followed by Šídák’s multiple comparisons test, comparing sham and ischemic animals within each time point. Quantitative data are presented as mean ± SD. Significant changes are indicated as ∗*p* < 0.05, ∗∗*p* < 0.01, ∗∗∗*p* < 0.001, and ∗∗∗∗*p* < 0.0001. Data are shown for *n* = 5 animals per group and time point. See also Figure S3; Table S1.

Because plasma LPC emerged as one of the most prominent late phospholipid changes after tMCAO, we next examined whether this reflected an isolated increase in LPC or a broader imbalance between LPC and PC pools (Figures 3A–3D). Plasma PC abundance showed a distinct temporal pattern (Figure 3A), whereas LPC abundance increased during post-ischemic progression (Figure 3B). At 7 days, LPC and PC changed in parallel, limiting the impact on the LPC/PC ratio. In the hippocampus, the LPC/PC ratio remained relatively stable across the post-ischemic period (Figure 3C). In contrast, at 4 months post-ischemia, plasma LPC showed a second increase, whereas PC did not increase proportionally and remained lower than in sham animals, resulting in a marked increase in the plasma LPC/PC ratio (Figure 3D). This divergence between plasma and hippocampal LPC/PC dynamics supports a tissue-specific remodeling pattern in which peripheral choline-containing phospholipids exhibit a more pronounced late imbalance than hippocampal phospholipid pools. The same hippocampal and plasma phospholipid species datasets, expressed as mol %, were analyzed with BioPAN to infer phospholipid interconversion reactions between lipid species with direct biochemical relationships within the LIPID MAPS pathway framework.^16^ In plasma, pathway analysis at 4 months post-ischemia indicated strong enrichment of inferred phospholipid interconversion reactions, with PC-to-LPC and LPC-to-PC transitions emerging as the most significant pathways (Figure 3E). According to BioPAN predictions, these reactions were predominantly associated with the release of saturated and monounsaturated fatty acids, particularly 16:0 and 16:1, alongside the preferential reacylation of longer-chain fatty acids, such as 20:0 and 22:5 (Figure 3F).

In the hippocampus, PC-LPC interconversion was comparatively less pronounced overall but showed increased activity at 7 days and again at 4 months post-ischemia according to BioPAN-inferred reaction scores (Figure 3G), indicating temporally restricted waves of fatty acid exchange within brain tissue. Collectively, these findings suggest that ischemia induces sustained phospholipid remodeling characterized by a tissue-specific LPC/PC imbalance and BioPAN-inferred acylation-deacylation dynamics, which reshape membrane composition and potentially influence the availability and redistribution of fatty acids with structural and signaling relevance.

### Post-ischemic phospholipid remodeling through bioinformatic stratification

To understand the impact of ischemia on membrane phospholipid remodeling, we applied a comprehensive bioinformatic and experimental approach focused on the Lands cycle and its implications at cellular, organellar, and biophysical levels. Based on the BioPAN-inferred phospholipid interconversion reactions described above, we focused on enzymes associated with acylation-deacylation reactions within the PC/LPC remodeling axis. These BioPAN-derived enzyme candidates were then analyzed using STRING to evaluate their functional interaction network. STRING analysis was performed using the full network option, evidence-based interaction edges, all active interaction sources, a medium confidence score of 0.400, and no additional first- or second-shell interactors. Additional information is provided in Table S2. The analysis began with an unsupervised hierarchical clustering of enzymes associated with these BioPAN-derived reactions; the resulting clusters were interpreted in the context of known phospholipid remodeling pathways. This clustering highlighted a major interaction module enriched in LPCAT, MBOAT, and PLA2-related enzymes, together with additional lipid-remodeling enzymes, such as PEMT and PISD (Figure S3A). Together, this network-based prioritization supported LPCAT, MBOAT, and PLA2-related enzymes as candidates for subsequent protein-level validation.

To contextualize these molecular signatures, we used multiple bioinformatic validations to explore potential neurobiological consequences. At the cellular level, a similarity analysis based on Pearson correlation was performed to assess how closely lipid responses under glutamate-induced stress in astrocyte, neuronal, and endothelial cultures resembled the hippocampal LPC/PC ratio dynamics observed at different post-ischemic time points (Figure S3C). For this purpose, a previously published phospholipidomic dataset derived from these coculture systems was used as a reference.^17^ During the early stage (6 h), hippocampal phospholipid profiles correlated more strongly with neurons (Figure S3C), while subacute post-ischemic phases (24 h–15 days) showed greater similarity to astrocyte-enriched signatures. In contrast, at 4 months post-ischemia, correlations shifted toward an endothelial-like phospholipidomic profile, consistent with late neurovascular remodeling.

Notably, although neuronal cultures showed partial concordance with hippocampal PC dynamics at 6 h and 1 month post-ischemia, they did not exhibit the pronounced reciprocal shift between PC and LPC observed in astroglial and endothelial systems. In astrocytes, PC levels increased while LPC decreased, resulting in a reduced LPC/PC ratio (Figures S3D–S3F). Conversely, endothelial phospholipid profiles displayed a marked decrease in PC accompanied by a reciprocal increase in LPC, leading to a significant elevation of the LPC/PC ratio at late stages (Figure S3D–S3F). These opposing patterns suggest that ischemia-induced phospholipid remodeling is cell-type specific, with astrocytes predominating during subacute repair phases and endothelial-associated phospholipid remodeling emerging during chronic stages, consistent with our previous observations in human cases of vascular dementia.^18^

To extend these lipidomic observations toward a biologically integrative interpretation, we mapped phospholipid class changes to subcellular compartments and membrane properties using LION/web analysis.^19^ This approach revealed a redistribution of phospholipids toward mitochondria, endoplasmic reticulum, and plasma membranes, accompanied by predicted alterations in membrane curvature, lateral diffusion, surface charge, and bilayer thickness (Figure S3C). These predictions support the notion that ischemia drives coordinated structural membrane adaptations through phospholipid remodeling networks, rather than stochastic fluctuations in individual lipid species.

Together, these analyses illustrate how integrative lipidomics and bioinformatic stratification can move beyond descriptive lipid profiling to infer candidate enzymatic nodes, cellular signatures, and membrane-level consequences of post-ischemic phospholipid remodeling. This framework provided a rationale for prioritizing Lands cycle-related enzymes for subsequent experimental validation.

### Region-specific validation of phospholipid remodeling enzymes after tMCAO

**Figure 4:**
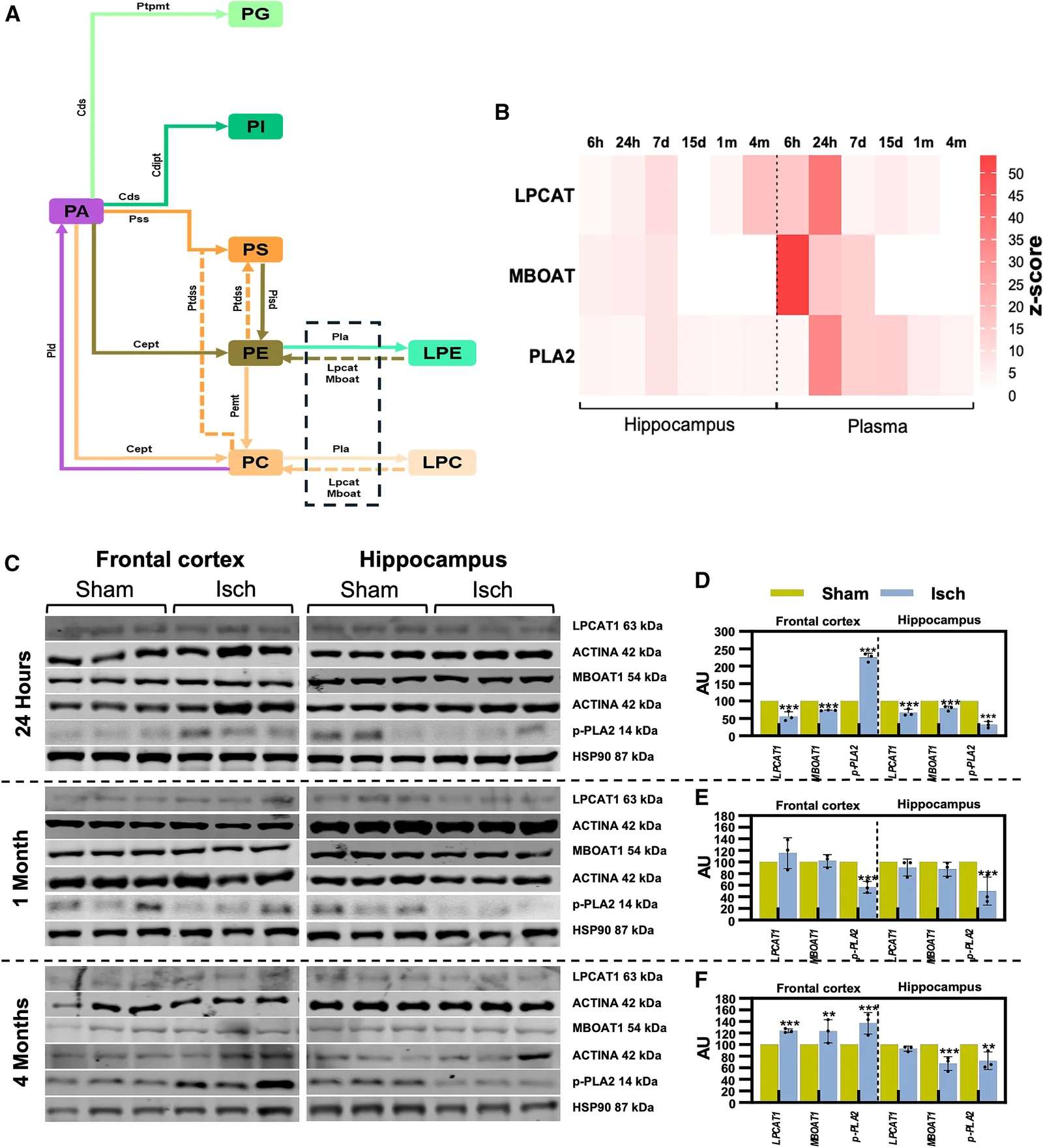
Protein dynamics of phospholipid remodeling enzymes after tMCAO (A) Schematic representation of the PC/LPC phospholipid remodeling axis and the participation of Land’s cycle enzymes in acylation-deacylation reactions. (B) BioPAN-derived summary of enzyme-associated phospholipid remodeling reactions identified across post-ischemic time points in hippocampus and plasma. (C) Representative western blot analysis of candidate phospholipid remodeling enzymes prioritized from BioPAN-derived reactions and STRING network analysis. (D–F) Quantification of protein levels at 24 h (D), 1 month (E), and 4 months (F) post-ischemia. The approximately 14-kDa band detected with the phospho-cPLA2 antibody was quantified consistently across samples; its molecular identity was not independently validated. LPCAT1 and MBOAT1 band intensities were normalized to actin, whereas phospho-cPLA2 band intensities were normalized to HSP90. For each protein, brain region, and post-ischemic time point, individual values were expressed relative to the mean of the corresponding sham group, which was set to 100%. Statistical comparisons were performed on the normalized individual animal values using multiple unpaired two-tailed Student’s *t* tests, assuming a single pooled variance across comparisons within each time point. No correction for multiple comparisons was applied, and α was set at 0.05. Quantitative data are presented as mean ± SD. Significant changes are indicated as ∗*p* < 0.05, ∗∗*p* < 0.01, ∗∗∗*p* < 0.001, and ∗∗∗∗*p* < 0.0001. Data are shown for *n* = 3 animals per group and time point. See also Figure S3; Table S2.

Based on the enzymes prioritized from BioPAN-derived reactions and STRING network analysis, we next validated the protein levels of key phospholipid remodeling enzymes in the hippocampus and frontal cortex. First, we schematized the PC/LPC remodeling axis and the participation of Lands cycle enzymes in phospholipid acylation-deacylation reactions (Figure 4A). We then summarized the BioPAN-derived temporal enrichment of these enzyme-associated reactions in the hippocampus and plasma, highlighting time-dependent remodeling patterns across post-ischemic stages (Figure 4B). The frontal cortex was selected because it is part of the infarcted territory and showed persistent mitochondrial dysfunction by TTC at 1 month post-ischemia. During the early post-ischemic stage (24 h), LPCAT1 and MBOAT1 protein levels were reduced in both the hippocampus and frontal cortex. In contrast, phosphorylated PLA2 (p-PLA2) exhibited a pronounced region-specific response, showing a marked increase in the cortex and a concomitant decrease in the hippocampus. This early divergence indicates that phospholipid deacylation-driven signaling is differentially regulated across brain regions immediately following ischemic injury.

At 1 month post-ischemia, LPCAT1 and MBOAT1 protein levels remained unchanged in both regions, while p-PLA2 levels were reduced in both the hippocampus and frontal cortex. This coordinated downregulation suggests a transient resolution of acute phospholipid signaling and inflammatory activity, consistent with the partial functional recovery observed at this stage. By 4 months, a region-specific divergence became evident. The frontal cortex maintained elevated levels of LPCAT1, MBOAT1, and p-PLA2, whereas in the hippocampus, MBOAT1 and p-PLA2 remained downregulated, while LPCAT1 levels remained unchanged.

Importantly, these findings indicate that enzyme protein levels alone do not fully account for phospholipid remodeling outcomes. Enzymatic activity is further shaped by substrate availability, coordinated action of complementary enzymes, post-translational regulation, and local membrane context. Together, this regional protein-level validation supports LPCAT1, MBOAT1, and p-PLA2 as temporally regulated components of the post-ischemic phospholipid remodeling response in hippocampal and cortical tissue, rather than isolated changes in individual enzymes.

### Differential expression of Lands cycle enzymes and lipid storage regulators in the post-ischemic brain

To extend the bioinformatic and lipidomic findings toward spatial resolution and given the dual role of Lands cycle enzymes in membrane remodeling and lipid storage regulation, we examined the spatiotemporal protein levels of key enzymes using *in situ*/tissue western immunoblotting. Analyses focused on LPCAT1, MBOAT1, and cPLA2, together with DGAT1 and SREBF2, which regulate neutral lipid synthesis and cholesterol homeostasis.^20^^,^^21^ All measurements were performed only in the ipsilateral ischemic hemisphere, at anterior (bregma +0.36 to −0.36) and medial (−2.64 to −3.12) levels according to the Paxinos atlas.^22^

We next examined whether lipid-regulatory protein changes showed anatomical specificity within the ipsilateral hemisphere. At 24 h post-ischemia, enzyme protein levels changed in a region-specific manner. In anterior sections, LPCAT1 and DGAT1 were reduced in cortical areas, whereas MBOAT1 and cPLA2 were markedly increased in the insular cortex, striatum, and septal nuclei (Figures S4C–S4F), indicating early engagement of phospholipid remodeling and lipid signaling pathways within and beyond the primary infarcted territory.

**Figure 5:**
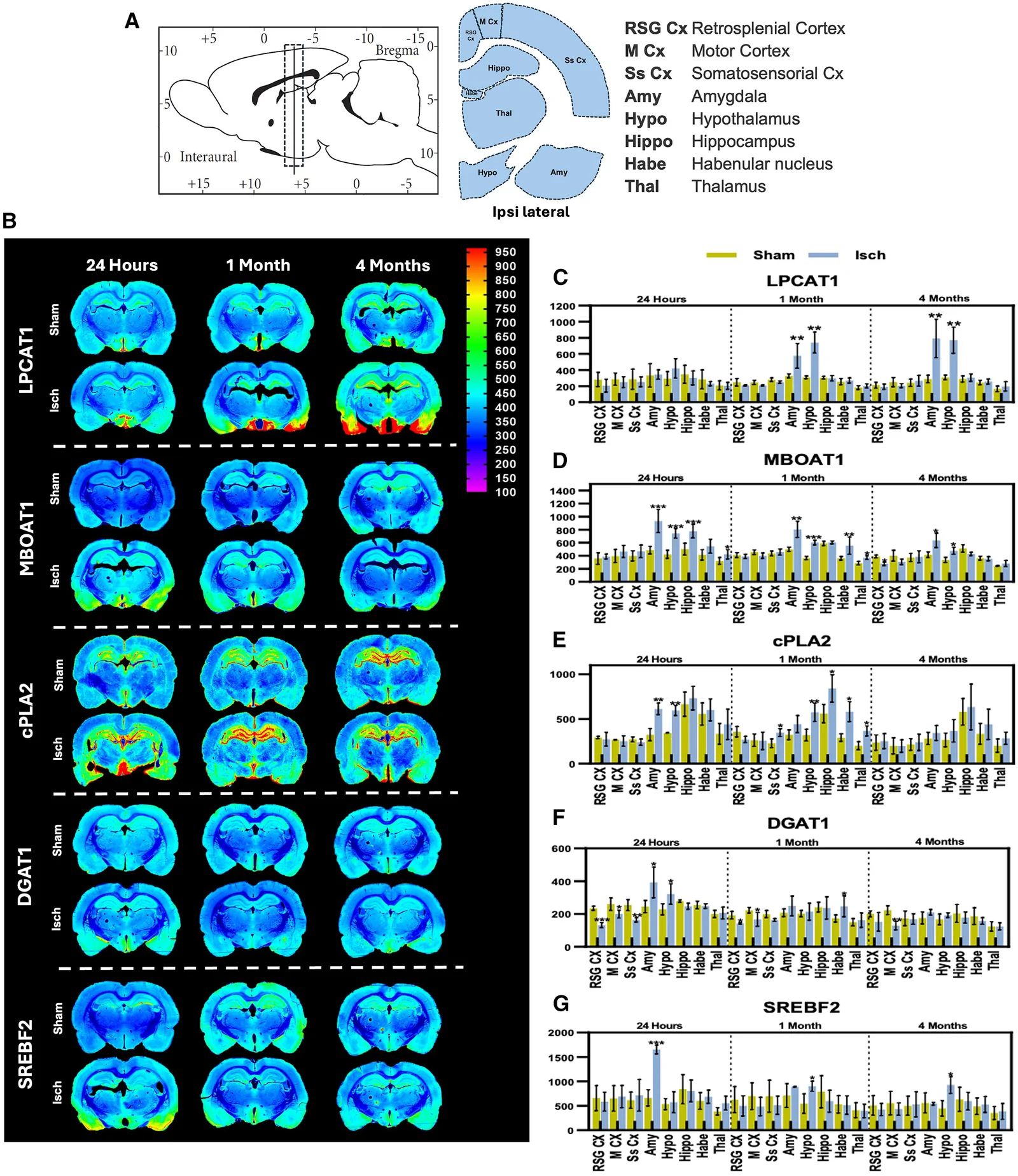
Regional protein levels of Lands cycle and lipid storage regulators at medial bregma levels after tMCAO (A) Schematic representation of the medial bregma level and analyzed ipsilateral brain regions. (B) Representative *in situ*/tissue western immunoblotting images showing protein signal distribution across post-ischemic time points. (C–G) Regional quantification of protein levels at 24 h, 1 month, and 4 months post-ischemia. Statistical analysis was performed using multiple unpaired two-tailed Student’s *t* tests comparing sham and ischemic animals within each brain region and time point. Quantitative data are presented as mean ± SD. Significant changes are indicated as ∗*p* < 0.05, ∗∗*p* < 0.01, and ∗∗∗*p* < 0.001. Data are shown for *n* = 3 animals per group and time point. See also Figure S4.

At medial levels, the amygdala and hypothalamus exhibited the most robust and persistent alterations (Figure 5A). MBOAT1 showed an early increase at 24 h that remained significant up to 4 months, while cPLA2 displayed a strong early induction followed by partial stability. In contrast, LPCAT1 increased predominantly at later stages (1 and 4 months), suggesting a delayed engagement of reacylation mechanisms during chronic remodeling. DGAT1 was transiently induced at 24 h, returning to sham-like levels thereafter, whereas SREBF2 remained elevated mainly in the hypothalamus, consistent with sustained lipid metabolic adaptation. Importantly, these findings indicate that the ipsilateral cortico-striatal infarct was accompanied by lipid-regulatory responses in ipsilateral exofocal regions, including limbic and hypothalamic territories, during chronic stages. In this study, the contralateral hemisphere was not analyzed.

Together, these findings integrate with the preceding phospholipidomic and bioinformatic analyses, reinforcing a model in which post-ischemic phospholipid reshaping reflects coordinated, region-specific, and time-dependent regulation of the Lands cycle. Rather than representing isolated molecular changes, the observed enzymatic patterns support a systems-level adaptation involving peri-infarct and vulnerable connected regions, lipid storage pathways, and membrane biophysical properties during long-term post-stroke remodeling.

### Temporal and regional accumulation of lipophilic structures in the post-ischemic brain

To characterize lipid-associated structures in the post-ischemic brain parenchyma, we employed three complementary lipophilic markers—boron-dipyrromethene (BODIPY), Sudan black, and oil red O—each exhibiting distinct chemical affinities for different lipid states. BODIPY/GFAP immunofluorescence was quantitatively analyzed in the anterior subventricular zone (SVZ)/periventricular region and olfactory tract (OT) of the ipsilateral hemisphere, supported by qualitative Sudan black and oil red O staining (Figures S5A and S5B). The differential staining patterns observed across markers indicate that ischemia induces heterogeneous lipid-associated structures rather than a single uniform lipid pool.

**Figure 6:**
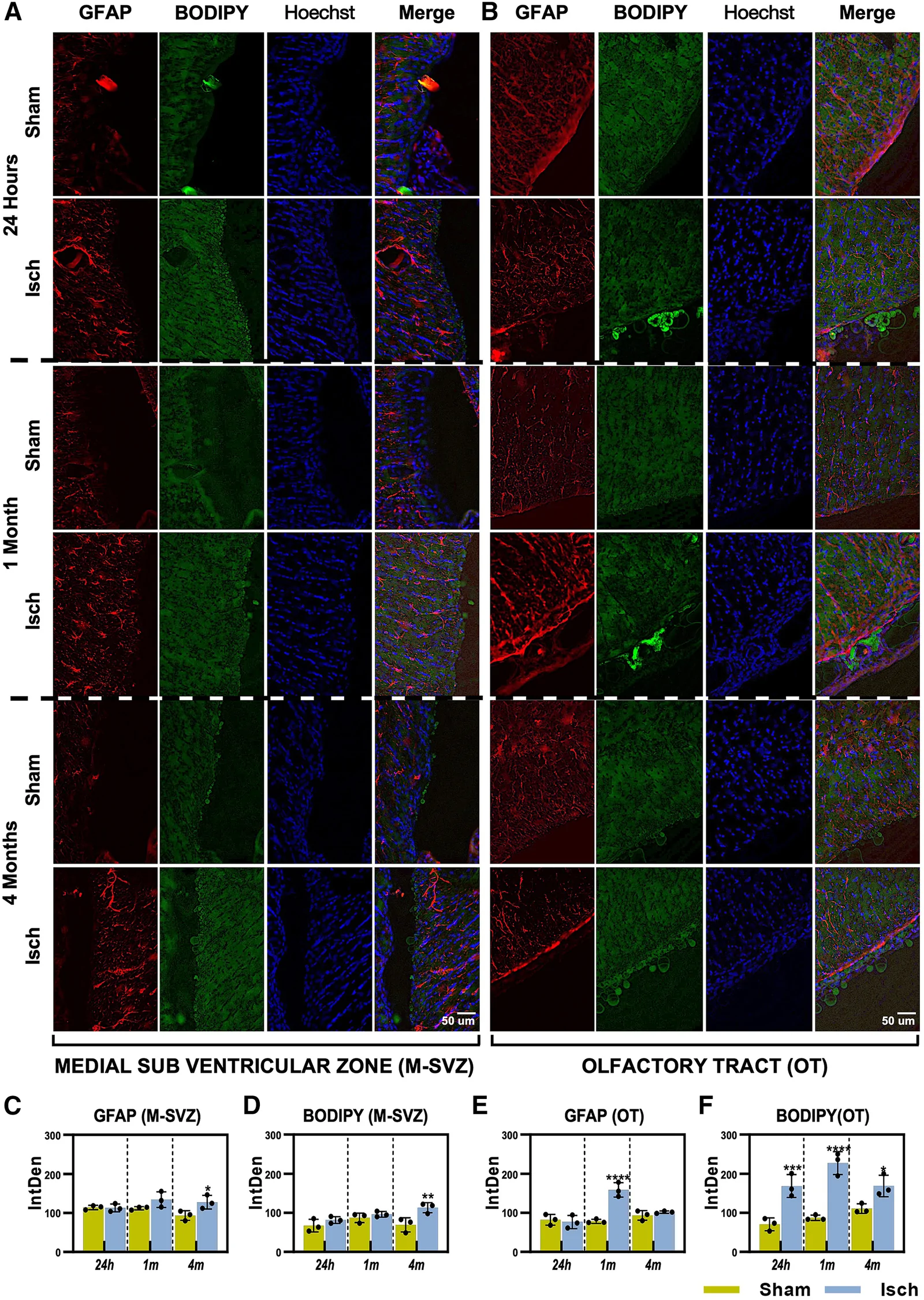
GFAP reactivity and BODIPY-positive lipophilic structures in the anterior SVZ/periventricular region and olfactory tract after tMCAO (A and B) Representative immunofluorescence images showing BODIPY 493/503, GFAP, and Hoechst labeling in the anterior lateral ventricle-associated SVZ/periventricular region (A) and olfactory tract (OT) (B) across post-ischemic time points. Scale bars: 50 μm. (C and D) Quantification of GFAP (C) and BODIPY 493/503 (D) signal in the anterior SVZ/periventricular region. (E and F) Quantification of GFAP (E) and BODIPY 493/503 (F) signal in the OT. GFAP and BODIPY signals were quantified as integrated density within predefined regions of interest. Statistical analysis was performed using ordinary two-way ANOVA followed by Šídák’s multiple comparisons test, comparing sham and ischemic animals within each time point. Quantitative data are presented as mean ± SD. Significant changes are indicated as ∗*p* < 0.05, ∗∗*p* < 0.01, ∗∗∗*p* < 0.001, and ∗∗∗∗*p* < 0.0001. Data are shown for *n* = 3 animals per group and time point. See also Figures S5 and S6.

BODIPY labeling revealed time- and region-dependent increases in lipid droplet (LD)-like structures in the SVZ and OT (Figures 6A–6F), accompanied by changes in GFAP immunoreactivity. Additional GFAP/BODIPY quantification revealed a lipophilic response in the amygdala detectable as early as 24 h (Figures S6A–S6C).

In complementary analyses, Sudan black preferentially labeled dense lipophilic material within the ischemic core at 24 h in the ipsilateral hemisphere, consistent with lipid-rich tissue alterations described after cerebral ischemia.^23^ Oil red O staining identified large lipid-rich structures from 24 h to 4 months (Figure S6E). These structures displayed well-defined, cell-like morphologies with process-bearing extensions, arguing against nonspecific dye precipitation and supporting their interpretation as lipid-rich cell-like profiles rather than amorphous lipid deposits.^24^ The size and morphology of the labeled structures varied across the three lipid dyes. While BODIPY, Sudan black, and oil red O are all widely used to detect neutral lipids, they highlighted partially different structures. These observations are descriptive and suggest that the chemical characteristics and lipid affinities of each marker may influence the patterns detected. Further mechanistic and compositional analyses will be required to clarify how these probes interact with distinct lipid pools in post-ischemic tissue.

Collectively, these findings indicate that cerebral ischemia induces temporally and regionally structured lipid-associated responses, with quantifiable GFAP and BODIPY alterations across cortical, limbic, periventricular, and OT regions. The differential detection by complementary lipophilic markers supports a model in which post-ischemic phospholipid reshaping gives rise to multiple lipid states and region-dependent lipophilic responses, rather than uniform lipid accumulation across the injured brain.

### Plasma lipid remodeling associates with central lipid dynamics and neurovascular alterations

**Figure 7:**
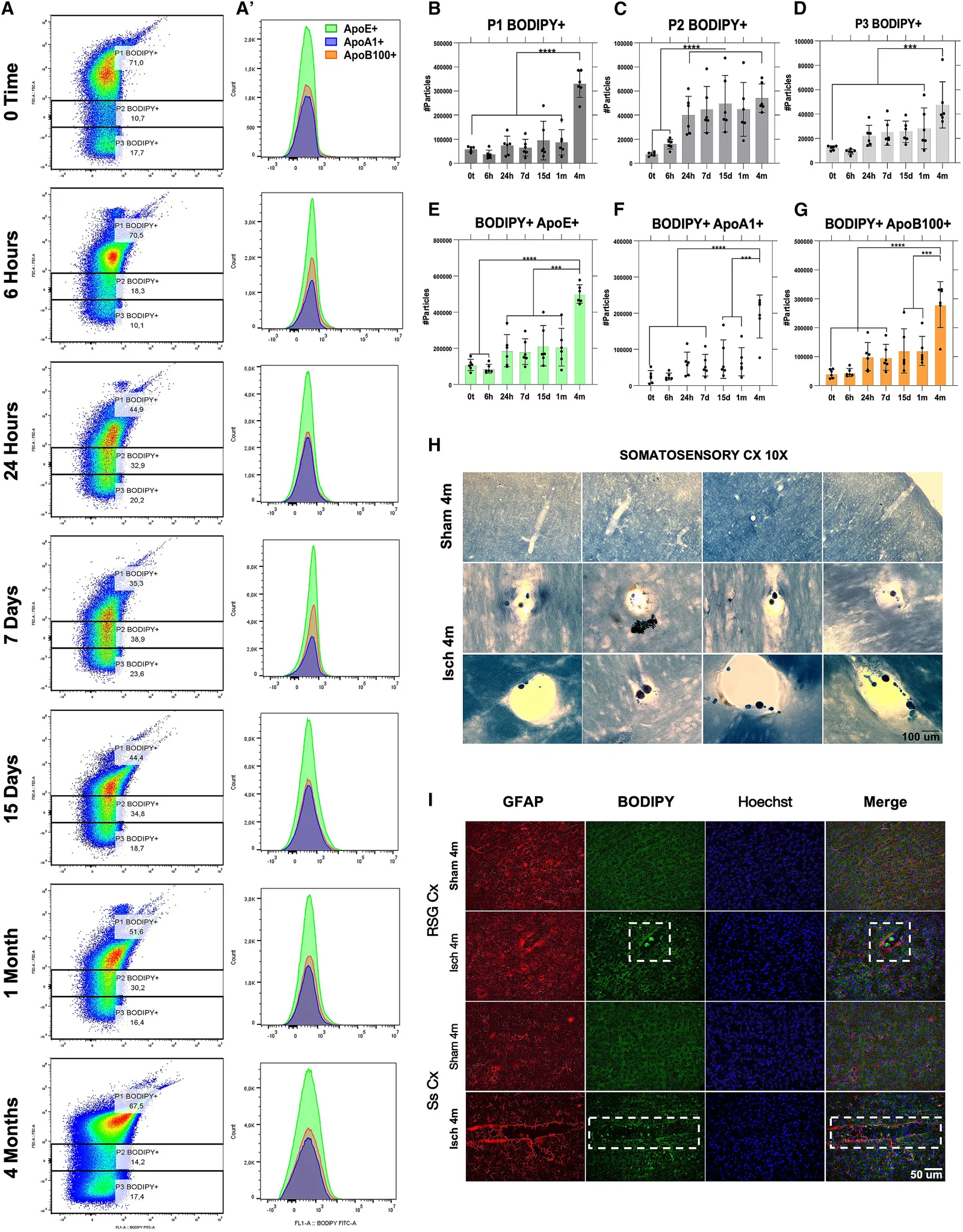
Longitudinal remodeling of circulating BODIPY^+^ lipophilic particles and qualitative vascular-associated lipid signals after tMCAO (A) Representative flow cytometry plots showing dynamic changes in BODIPY^+^ P1, P2, and P3 particle populations, discriminated by size, together with representative gating for ApoE-, ApoA1-, and ApoB100-associated BODIPY^+^ particles at 0 h, 6 h, 24 h, 7 days, 15 days, 1 month, and 4 months post-ischemia. (B–D) Quantification of circulating BODIPY^+^ P1 (B), P2 (C), and P3 (D) particle populations. (E–G) Quantification of ApoE-associated (E), ApoA1-associated (F), and ApoB100-associated (G) BODIPY^+^ particles. (H) Representative qualitative Sudan black images showing vascular-like profiles at chronic post-ischemic stages. Scale bars: 100 μm. (I) Representative qualitative BODIPY 493/503, GFAP, and Hoechst images showing lipophilic signal adjacent to GFAP-positive perivascular-like structures in post-ischemic brain tissue. Scale bars: 50 μm. Flow cytometry data were analyzed using one-way ANOVA followed by Tukey’s multiple comparisons test. Quantitative data are presented as mean ± SD. Significant changes are indicated as ∗*p* < 0.05, ∗∗*p* < 0.01, ∗∗∗*p* < 0.001, and ∗∗∗∗*p* < 0.0001. Flow cytometry analyses were performed with *n* = 6 animals followed longitudinally. Histological images are representative qualitative observations selected from *n* = 3 animals and were not used for quantitative comparisons or direct vascular identification.

To explore whether cerebral lipid remodeling, including alterations in phospholipid balance reflected by an increased plasma LPC/PC ratio, is accompanied by systemic lipid changes, we performed longitudinal flow cytometry analyses to characterize circulating BODIPY^+^ lipophilic particles associated with apolipoproteins. At baseline (0 h), three BODIPY^+^ particle populations were defined based on size (P1–P3) (Figure 7A). Following ischemia, the three BODIPY^+^ particle populations displayed distinct temporal trajectories (Figures 7B–7D). The intermediate P2 population increased significantly from 24 h onward, whereas P1 and P3 showed more limited changes at intermediate time points followed by a pronounced increase at 4 months post-ischemia. Thus, rather than a sustained redistribution between particle populations, the longitudinal profile indicates progressive remodeling of circulating BODIPY^+^ particles, with the strongest changes observed at the chronic 4-month stage.

In parallel, analysis of BODIPY^+^ particle association with ApoE, ApoA1, and ApoB100 revealed increasing particle-associated signals over the post-ischemic period, with the most pronounced and statistically significant elevations observed at 4 months post-ischemia (Figures 7E–7G). Although these analyses are correlational in nature, the temporal convergence of systemic lipophilic particle remodeling with central lipid alterations supports the concept of coordinated lipid dynamics rather than isolated compartmental changes.

Notably, the systemic increase in circulating lipophilic particles coincided temporally with vascular-like profiles and lipophilic signals adjacent to GFAP-positive perivascular-like structures at 4 months post-ischemia (Figures 7H and 7I). Together, these findings suggest that chronic post-ischemic lipid remodeling involves interconnected central and peripheral processes potentially associated with long-term neurovascular remodeling and sustained reshaping of systemic lipophilic particles.

## Discussion

We identify a delayed trajectory of post-ischemic functional vulnerability, characterized by late neurological deterioration and cognitive impairment, associated with a persistent imbalance between brain and plasma phospholipid pools. Integrated lipidomic profiling, pathway-level bioinformatic analysis, and targeted protein analyses converge to reveal sustained phospholipid remodeling with defined temporal progression and regional specificity, supporting ongoing brain-periphery lipid dysregulation beyond the acute injury phase. The primary cortical-striatal ischemic lesion decreased over time, whereas late functional and lipid-associated alterations persisted, consistent with intrinsic remodeling of vulnerable post-ischemic networks. These findings extend prior evidence linking plasma phospholipid alterations to PSCI^7^^,^^25^ and demonstrate that lipid remodeling persists into the chronic post-ischemic stage, highlighting its potential contribution to long-term functional vulnerability, which was also reflected in plasma lipid alterations.

Our data support a coordinated remodeling program in which central and peripheral lipid dynamics evolve in parallel over time. Among the implicated pathways, the Lands cycle emerged as a recurrent axis, supported by BioPAN-inferred, compartment-specific PC/LPC interconversion patterns in the hippocampus and plasma after ischemia. These pathway-level predictions were supported by experimental validation of key remodeling enzymes, including cPLA2, LPCAT1, and MBOAT1, particularly in vulnerable ipsilateral regions such as the amygdala and hypothalamus, which also displayed astrogliosis and lipophilic signal alterations. In parallel, a divergence in the LPC/PC ratio between hippocampus and plasma, together with increased association of circulating lipophilic particles with ApoE, ApoA1, and ApoB100, points to a persistent systemic lipid imbalance during the chronic post-ischemic phase.

A central finding is the late longitudinal divergence in the LPC/PC ratio between the hippocampus and plasma, indicating sustained differences between central and peripheral phospholipid remodeling. In line with this long-term pattern, we previously reported that an increased plasma LPC/PC ratio at 1 month post-ischemia correlates with cognitive impairment and hippocampal structural alterations, including astrogliosis, inflammation, and loss of cell adhesion.^7^ Earlier time points have been explored; experimental stroke studies in animals have described rapid changes in LPC and PC distribution as early as 24 h post-ischemia, including divergent alterations between brain parenchyma and plasma and significant shifts in circulating LPC levels.^26^^,^^27^ In contrast, experimental stroke models in mice show that an early increase in brain LPC/PC is associated with functional recovery during the first weeks after ischemia.^28^ Clinical studies in post-stroke patients have identified plasma LPC and PC alterations associated with functional recovery, underscoring the translational relevance of systemic phospholipid remodeling after ischemia.^29^ By extending the temporal window to 1–4 months, our data reveal a distinct late-phase lipid remodeling pattern, characterized by nearly stable hippocampal LPC/PC levels and a marked increase in plasma LPC/PC. Importantly, this systemic shift temporally overlaps with late neurological deterioration and cognitive impairment, supporting a transition from an early adaptive response to a later systemically coordinated process.

At the mechanistic level, the observed LPC/PC dynamics are consistent with temporal engagement of the Lands cycle, a central phospholipid remodeling pathway that regulates membrane repair, fatty acid recycling, and inflammatory signaling.^28^ Within this framework, cPLA2-mediated deacylation promotes the release of polyunsaturated fatty acids, such as arachidonic acid and DHA, which modulate inflammatory and resolution pathways.^30^ Reacylation by LPCAT and MBOAT enzymes restores phospholipid pools and reshapes membrane composition.^31^ Our BioPAN analysis indicates coordinated enrichment of PLA2- and LPCAT-related reactions in both hippocampus and plasma, suggesting shared remodeling logic across compartments. Importantly, enzyme expression does not equate to enzymatic activity, and our conclusions rely on integrated lipidomic-bioinformatic-protein concordance rather than direct functional manipulation. However, our previous studies and the cellular similarity analysis presented here support a potential functional association. In endothelial cells exposed to glutamate toxicity, an increased LPC/PC ratio was accompanied by disruption of cell adhesion. *In vivo*, silencing of a gene involved in post-ischemic inflammation reduced neurological impairment together with p-cPLA2 levels.^17^ In neuronal cells, inhibition or silencing of cPLA2 prevented glutamate-induced tau hyperphosphorylation.^30^

Beyond membrane remodeling, Lands cycle enzymes are increasingly recognized as regulators of LD biogenesis and membrane curvature, linking phospholipid turnover to neutral lipid storage.^32^^,^^33^^,^^34^ Consistent with this, we observed temporal regulation of DGAT1 and sustained upregulation of SREBF2, a master regulator of cholesterol and lipid biosynthesis.^20^^,^^21^ LD accumulation following ischemia has been extensively documented, particularly in microglia during acute and subacute phases.^35^ However, the mechanisms governing LD clearance and long-term fate remain incompletely defined. Autophagy and lipophagy have been proposed as key pathways regulating LD turnover and limiting lipotoxicity.^36^^,^^37^

In parallel, lipid clearance linked to phagocytosis of membrane debris and myelin-derived cargo is increasingly recognized as a critical component of post-ischemic tissue remodeling.^38^^,^^39^^,^^40^^,^^41^ Microglia undergoing phagocytosis display transcriptional programs enriched in lipid metabolism and cholesterol handling following stroke,^42^ while astrocytes can remain reactive for several months after ischemia,^43^ consistent with the increased GFAP reactivity detected in several vulnerable regions at 4 months post-ischemia, potentially sustaining lipid remodeling during chronic stages.

A major strength of the present study is the demonstration that late cerebral lipid remodeling is paralleled by systemic lipid changes, particularly the accumulation of neutral lipids and lipophilic particles in plasma at 4 months post-ischemia. This observation is consistent with the broader need to redistribute poorly metabolizable lipids to limit cellular lipid accumulation.^44^^,^^45^ HDL-mediated lipid transport provides a plausible mechanism linking the CNS to systemic circulation,^46^^,^^47^^,^^48^ with apolipoproteins, such as ApoE and ApoA1, facilitating lipid trafficking and clearance.^48^^,^^49^^,^^50^

Spatially, we observed lipophilic material accumulating in periventricular regions and the OT, areas previously implicated in CSF drainage and glymphatic-associated clearance pathways, suggesting that alterations in fluid-mediated lipid transport may contribute to the observed spatial redistribution.^51^^,^^52^^,^^53^ While these observations are correlative, they support the concept of a spatially organized lipid redistribution process, rather than nonspecific diffuse parenchymal accumulation. In this context, the concept of “lipid clearance” is used to describe the coordinated emergence of sustained cerebral lipid remodeling alongside measurable shifts in plasma lipid profiles. Rather than implying a direct or singular transport pathway from brain to blood, this framework reflects a systems-level coupling between central lipid metabolism, lipoprotein-associated lipid handling, and peripheral lipid dynamics, which becomes evident during chronic post-ischemic stages.^44^^,^^51^^,^^54^

Consistent with this framework, the redistribution of BODIPY^+^ particles and their association with ApoE, ApoA1, and ApoB100 resemble key features of reverse cholesterol transport.^48^^,^^55^ ApoB100 mediates lipid delivery in LDL particles,^50^ whereas ApoA1 supports lipid efflux through HDL-associated pathways.^49^ ApoE, a central regulator of brain lipid metabolism, modulates cerebrovascular vulnerability and recovery after ischemia.^50^^,^^56^ In this context, the vascular-like profiles and lipophilic signals adjacent to GFAP-positive perivascular-like structures observed at late stages may further contribute to persistent neurovascular dysfunction and impaired lipid handling.^57^^,^^58^

In conclusion, the principal contribution of this work lies in linking late post-ischemic functional vulnerability to a prolonged, coordinated brain-plasma lipid remodeling program unfolding across chronic post-ischemic stages. The proposed lipid clearance framework provides a unifying integrative conceptual model that integrates lipidomic, bioinformatic, cellular, and vascular observations across an extended post-ischemic timeline.

### Limitations of the study

Importantly, we frame the interpretative model as correlative and hypothesis driven, and we acknowledge that targeted mechanistic studies will be required to define the precise cellular sources, transport routes, and regulatory checkpoints involved. BioPAN outputs represent inferred pathway activity, and changes in protein abundance do not establish enzymatic activity or causal involvement. Regional histological and protein analyses were performed with modest sample sizes and were restricted to the ipsilateral hemisphere, precluding the assessment of hemispheric asymmetry. In addition, only adult male rats were studied; therefore, sex-dependent responses and generalizability to females remain unknown. Nevertheless, the consistent association between cerebral lipid remodeling and plasma lipid signatures supports the potential of peripheral phospholipids as non-invasive biomarkers of chronic post-stroke vulnerability and provides a framework for future studies on endogenous lipid clearance and therapeutic modulation. Future studies combining lipid tracing, functional manipulation of remodeling enzymes, particle characterization, and analyses in both sexes will be required to test the proposed mechanisms and their translational relevance.

## Resource availability

### Lead contact

Further information and requests for resources and reagents should be directed to and will be fulfilled by the lead contact, Gloria Patricia Cardona-Gómez patricia.cardonag@udea.edu.co.

### Materials availability

This study did not generate new, unique reagents.

### Data and code availability

• The phospholipidomic datasets generated in this study have been deposited at Zenodo (https://doi.org/10.5281/zenodo.18675043) and are publicly available as of the date of publication. The accession number is listed in the key resources table.
• This paper does not report original code.
• Any additional information required to reanalyze the data reported in this paper is available from the lead contact upon reasonable request.

## Acknowledgments

This study was supported by grants from 10.13039/100022965Minciencias and 10.13039/501100014908ICETEX # 82336 (G.P.C.-G.), CODI UdeA (G.P.C.-G.). The lipid analyses described in this work were performed at the Kansas Lipidomics Research Center Analytical Laboratory. Instrument acquisition and lipidomics method development were supported by the 10.13039/100000001National Science Foundation (including support from the Major Research Instrumentation program; most recent award DBI-1726527), K-IDeA Networks of Biomedical Research Excellence (INBRE) of the National Institutes of Health (P20GM103418), USDA 10.13039/100005825National Institute of Food and Agriculture (Hatch/Multi-State project 1013013), and Kansas State University.

## Author contributions

I.D.S.-C., conceptualization, data analysis, investigation, discussion, experimentation, formal analysis, data curation, writing – original draft; J.G.-V., experimentation, writing – review and editing; G.P.C.-G., conceptualization, data analysis, investigation, discussion, supervision, funding acquisition, writing – original draft, writing – review, and editing.

## Declaration of interests

The authors declare no competing interests.

## Declaration of generative AI and AI-assisted technologies in the writing process

During the preparation of this work, the authors used ChatGPT (OpenAI) to support language editing, manuscript organization, and consistency review. After using this tool, the authors reviewed and edited the content as needed and take full responsibility for the content of the publication.

## STAR★Methods

### Key resources table

**Table:** 

REAGENT or RESOURCE	SOURCE	IDENTIFIER
**Antibodies**		

Rabbit polyclonal anti-LPCAT1	Invitrogen/Thermo Fisher Scientific	Cat# PA5-106278; RRID: AB_2853955
Rabbit polyclonal anti-MBOAT1	MyBioSource	Cat# MBS9236794
Rabbit polyclonal anti-phospho-cPLA2 (Ser505)	Cell Signaling Technology	Cat# 2831S; RRID: AB_2164445
Mouse monoclonal anti-actin (clone ACTN05/C4)	Invitrogen/Thermo Fisher Scientific	Cat# MA5-11869; RRID: AB_11004139
Mouse monoclonal anti-HSP90 (clone AC88)	Assay Designs	Cat# SPA-830; lot 12010801; RRID: AB_10616102
Mouse monoclonal anti-GFAP (clone GA5)	eBioscience/Thermo Fisher Scientific	Cat# 14–9892-82; RRID: AB_10598206
Mouse anti-cPLA2	Santa Cruz Biotechnology	Cat# sc-454; RRID: AB_627288
Rabbit polyclonal anti-DGAT1	Invitrogen/Thermo Fisher Scientific	Cat# PA5-117074; RRID: AB_2901704
Rabbit anti-SREBF2/SREBP2	LifeSpan BioSciences	Cat# LS-B1609
Rabbit recombinant monoclonal anti-ApoA1 (clone 19H20L19)	Invitrogen/Thermo Fisher Scientific	Cat# 701239; RRID: AB_2532437
Mouse monoclonal anti-Apolipoprotein B (clone F2C9)	Invitrogen/Thermo Fisher Scientific	Cat# MIA1609; RRID: AB_11152638
PE anti-ApoE antibody (clone E6D7)	BioLegend	Cat# 803404; RRID: AB_2801139
Goat anti-Mouse IgG (H + L), Alexa Fluor 594	Invitrogen/Thermo Fisher Scientific	Cat# A-11005; RRID: AB_2534073
IRDye 800CW Goat anti-Rabbit IgG (H + L)	LI-COR Biosciences	P/*N* 926–32211; RRID: AB_621843
IRDye 680RD Goat anti-Mouse IgG	LI-COR Biosciences	P/*N* 926–68070; RRID: AB_10956588
Goat anti-Rabbit IgG (H + L), Alexa Fluor 405	Invitrogen/Thermo Fisher Scientific	Cat# A-31556; RRID: AB_221605
Goat anti-Mouse IgG (H + L), Alexa Fluor 647	Invitrogen/Thermo Fisher Scientific	Cat# A-21235; RRID: AB_2535804

**Biological samples**		

Rat plasma	This paper	Act No. 136, November 17, 2020
Rat hippocampal tissue	This paper	Act No. 136, November 17, 2020
Rat frontal cortex tissue	This paper	Act No. 136, November 17, 2020
Perfusion-fixed rat brain tissue/sections	This paper	Act No. 136, November 17, 2020

**Chemicals, peptides, and recombinant proteins**		

BODIPY 493/503	Invitrogen/Thermo Fisher Scientific	Cat# D3922
Hoechst 33342 nucleic acid stain	Invitrogen/Thermo Fisher Scientific	Cat# H3570

**Deposited data**		

Hippocampal and plasma phospholipidomic datasets	This paper; Zenodo	Zenodo: https://doi.org/10.5281/zenodo.18675043
Previously published astrocyte, neuron, and endothelial coculture phospholipidomic dataset	Bedoya-Guzmán et al.17	https://doi.org/10.3389/fnagi.2023.1194203

**Experimental models: Organisms/strains**		

Rat: Wistar albino, male, 3 months old (400–450 g)	SIU vivarium, University Research Center, University of Antioquia	Act No. 136, November 17, 2020

**Software and algorithms**		

ImageJ (version not specified)	National Institutes of Health	https://imagej.net/ij/
Image Studio Software v6.0	LI-COR Biosciences	https://www.licor.com/bio/image-studio
GraphPad Prism 8	GraphPad Software, LLC	https://www.graphpad.com/scientific-software/prism/
FlowJo v10	FlowJo, LLC/BD Biosciences	https://www.flowjo.com/solutions/flowjo
BioPAN	LIPID MAPS; Gaud et al.16	https://www.lipidmaps.org/biopan
LipidSig web platform	BioinfOMICS	https://lipidsig.bioinfomics.org/
STRING	STRING Consortium	https://string-db.org/
LION/web	Molenaar et al.19	https://lipidontology.com/

**Other**		

API 4000 Q-TRAP detection system (4000 QTRAP LC-MS/MS System)	SCIEX	Model: 4000 QTRAP; https://sciex.com/products/mass-spectrometers/qtrap-systems/qtrap-4000-system
Mini-PROTEAN electrophoresis system	Bio-Rad Laboratories	https://www.bio-rad.com/en-us/product/mini-protean-tetra-vertical-electrophoresis-cell
Odyssey Infrared Imaging System	LI-COR Biosciences	https://www.licor.com/bio/odyssey-family
IX81 inverted microscope	Olympus (now Evident Scientific)	Model: IX81; https://evidentscientific.com/en/discontinued-products
CytoFLEX flow cytometer	Beckman Coulter Life Sciences	Model: CytoFLEX; https://www.beckman.com/flow-cytometry/research-flow-cytometers/cytoflex

### Experimental model and study participant details

#### Animals

All animal procedures were conducted in accordance with the ARRIVE guidelines, the Guide for the Care and Use of Laboratory Animals, 8th edition, published by the National Institutes of Health, and Colombian regulations, including Law 84/1989 and Resolution 8430/1993. Experimental protocols were approved by the Animal Experimentation Ethics Committee of the University of Antioquia, Medellín, Colombia under ethical approval number Act No. 136 (extraordinary session, November 17, 2020).

Male Wistar albino rats were obtained from the pathogen-free colony maintained at the SIU vivarium, University Research Center, University of Antioquia, Medellín, Colombia. Animals were housed under a 12 h light/dark cycle with *ad libitum* access to food and water. All efforts were made to minimize animal suffering and reduce the number of animals used. Only male rats were included in this study; therefore, sex-dependent effects were not evaluated.

Two independent cohorts of three-month-old male rats, weighing 400–450 g, were used. Animals were randomly assigned to sham or ischemic groups. One ischemic animal died during follow-up, corresponding to a mortality of 1.9% among ischemic animals (1 of 54); no deaths occurred in sham-operated animals. The final sample sizes reported in each figure legend reflect the animals included in each analysis.

First cohort. Animals were assigned to the experimental time points at 6 h, 24 h, 7 d, 15 d, 1 month, and 4 months post-ischemia. Six animals per experimental group were included for longitudinal neurological and motor assessments at each time point. Morris water maze acquisition was evaluated at 1 and 4 months, with *n* = 5 animals per group at both time points. Hippocampal and plasma lipidomic analyses were performed in a subset of five animals per group and time point.

TTC subgroup. A subset of animals from the first cohort was used for TTC staining and infarct quantification at 24 h, 1 month, and 4 months post-ischemia. Four ischemic animals were analyzed at each time point, and sham control tissue was included at 24 hours.

Second cohort. Six animals per experimental group were evaluated at 24 h, 1 month, and 4 months post-ischemia. Animals assigned to the 4-month endpoint underwent longitudinal tail-blood collection while alive, before surgery (0 h) and at 6 h, 24 h, 7 d, 15 d, 1 month, and 4 months post-ischemia, for flow cytometry analysis of circulating BODIPY^+^ lipophilic particles and apolipoprotein-associated events. At each terminal time point, three animals per group were perfused and their brains were fixed for histological staining, immunofluorescence, and *in situ*/tissue Western immunoblotting analyses. The remaining three animals per group were not perfused, and their brains were rapidly frozen and stored at −80°C for conventional Western blot analysis.

### Method details

#### Transient middle cerebral artery occlusion (tMCAO)

Rats were anesthetized with ketamine (60 mg/kg) and xylazine (5 mg/kg), followed by inhalation anesthesia using 2–4% isoflurane in 96% oxygen. The right common carotid artery was exposed, and a nylon filament of 0.26 mm diameter was advanced approximately 17 mm into the internal carotid artery to occlude the origin of the middle cerebral artery. After 60 min of occlusion, the filament was withdrawn to allow reperfusion. Sham-operated animals received the same anesthetic regimen and underwent artery exposure without middle cerebral artery occlusion. All surgeries were performed at room temperature.

According to the experimental design shown in Figure 1A, neurological scoring and sample collection were performed at predefined time points. At each corresponding terminal time point, all animals were euthanized in a CO_2_ chamber. Immediately after euthanasia, blood was collected by cardiac puncture into anticoagulant tubes, centrifuged at 3600 rpm for 5 min to obtain plasma, and stored at −80°C. In the first cohort, brains designated for lipidomic analyses were isolated, frozen on dry ice, and stored at −80°C until processing.

In the second cohort, animals were euthanized in a CO_2_ chamber at 24 h, 1 month, and 4 months post-ischemia. Immediately after euthanasia, blood was collected by cardiac puncture into anticoagulant tubes, centrifuged at 3600 rpm for 5 min to obtain plasma, and stored at −80°C. Following blood collection, three animals per group were transcardially perfused with 4% paraformaldehyde for histological staining, immunofluorescence, and *in situ*/tissue Western immunoblotting analyses. The remaining three animals per group were not perfused; their brains were directly removed, rapidly frozen, and stored at −80°C for conventional Western blot analysis.

#### Determination of infarct volume

Animals assigned to TTC analysis were euthanized in a CO_2_ chamber at 24 h, 1 month, and 4 months after tMCAO reperfusion. Brains were carefully removed, cooled in phosphate-buffered saline for 5 min, and sliced coronally every 2 mm using a rat brain matrix. Slices were stained with 0.5% 2,3,5-triphenyltetrazolium chloride in physiological buffer for 30 min at 37°C in the dark, washed twice with saline, fixed with 4% paraformaldehyde for 30 min at room temperature, and imaged for analysis.

Infarct size was measured using ImageJ software. Infarct areas were integrated across serial sections to calculate infarct volume. The total volume of each hemisphere and the infarcted volume were determined by integrating areas across slices. Infarct volume was corrected to compensate for swelling in the ischemic hemisphere and expressed as a percentage of the contralateral hemisphere volume.

#### Neurological assessment

Neurological performance was evaluated at 6 h, 24 h, 7 d, 15 d, 1 month, and 4 months post-ischemia according to the experimental timeline. Neurological function was scored using an 18-point system previously applied by García et al. The assessment included six tests: spontaneous activity, symmetry in limb movement, forelimb extension, climbing, body proprioception, and response to vibrissae touch. Each test was scored on a 0–3 scale, with a maximum total score of 18 indicating normal neurological function. Evaluations were performed at each predefined time point in the same order and at the same time for all animals.

#### Inclined plane test

Motor/postural performance was evaluated using the inclined plane test at 6 h, 24 h, 7 d, 15 d, 1 month, and 4 months post-ischemia. The maximum angle at which the rat could maintain its position for 10 s was recorded as the final angle and used as a measure of functional impairment.

#### Morris water maze

Spatial learning was assessed at 1 month and 4 months post-ischemia using the Morris water maze. Animals were trained to find a submerged, non-visible platform in a circular pool. During the acquisition phase, four trials per day were conducted over seven consecutive days. In each trial, animals were placed at different starting points and allowed to swim until they located the platform. Animals failing to locate the platform within 60 s were guided to it and allowed to remain there for 10–15 s. Escape latency, defined as the time required for each animal to locate the hidden platform, was recorded in each trial as a measure of spatial learning acquisition. Over the training days, a decrease in escape latency was expected, indicating improved acquisition of the platform location.

#### Lipid extraction

Total lipids were extracted from the hippocampus and plasma using the Folch method with chloroform: methanol (2:1, v/v) supplemented with 0.005% butylated hydroxytoluene. Hippocampal tissue was homogenized in the extraction solvent, followed by phase separation with 0.9% NaCl. Samples were centrifuged at 3000 rpm for 3 min, and the organic phase was collected in glass tubes. Solvents were evaporated, extracts were lyophilized to remove residual moisture, and lipid composition was analyzed by mass spectrometry. Lipid abundances were expressed as molar percentage (%mol) for class- and species-level analyses, and selected plasma PC and LPC abundances were also reported as nmol/mL.

#### Mass spectrometry

Lipidomic data were acquired using an automated ESI-MS/MS approach at the Kansas Lipidomics Research Center using an API 4000 Q-TRAP detection system, as previously described. Lipid species were identified according to mass-to-charge ratios and quantified relative to internal standards. Internal standards included 0.30 nmol lysoPG 14:0, 0.30 nmol lysoPG 18:0, 0.30 nmol di14:0 PG, 0.30 nmol 14:0 lysoPE, 0.30 nmol 18:0 lysoPE, 0.60 nmol 13:0 lysoPC, 0.60 nmol 19:0 lysoPC, 0.60 nmol di12:0 PC, 0.60 nmol di24:1 PC, 0.30 nmol 14:0 lysoPA, 0.30 nmol 18:0 lysoPA, 0.30 nmol di14:0 PA, 0.30 nmol di20:0 phytanoyl PA, 0.20 nmol di14:0 PS, 0.20 nmol diPhy PS, 0.28 nmol 16:0e18:0 PI, and 0.10 nmol di18:0 PI. The system detected phospholipid classes and their respective molecular species, identified by total carbon number and degree of unsaturation. Lipid concentrations were normalized to the molar concentration of all species in each sample, and final data were expressed as average molar percentage (%mol) values.

#### Bioinformatic analyses

Bioinformatic analyses were performed using the hippocampal and plasma phospholipidomic datasets generated in this study. Lipid abundances were expressed as molar percentage (%mol), and ischemic samples were compared with their corresponding time-matched sham controls at each post-ischemic stage. Before platform-specific analyses, lipid species with zero or missing values were handled according to each platform’s requirements, and lipid nomenclature was checked and harmonized to match platform-compatible formats.

Phospholipid interconversion reactions were inferred using BioPAN, a LIPID MAPS web-based tool for pathway-oriented analysis of lipidomics datasets. BioPAN was used to identify lipid species with direct biochemical relationships and to infer class- and species-level phospholipid remodeling reactions between ischemic and sham groups. BioPAN outputs were interpreted as pathway-level reaction scores and inferred remodeling patterns rather than direct measurements of enzymatic activity. Z-scores generated by BioPAN were used to represent the standardized magnitude and direction of inferred lipid reaction changes relative to the corresponding sham control group.

LipidSig was used for species-level visualization and exploratory multivariate analyses, including hierarchical clustering and principal component analysis. Hierarchical clustering was performed using Pearson correlation, and heatmap colors represented relative abundance patterns within the clustered dataset rather than direct log_2_ fold-change values. PCA was used to identify lipid species contributing most strongly to group separation across post-ischemic time points.

Candidate enzymes associated with BioPAN-inferred phospholipid remodeling reactions were analyzed using STRING to evaluate their functional interaction network. STRING analysis was performed using the full network option, evidence-based interaction edges, all active interaction sources, a medium confidence score of 0.400, and no additional first- or second-shell interactors. STRING-derived clusters were used to prioritize enzyme groups associated with phospholipid acylation–deacylation reactions, particularly LPCAT, MBOAT, and PLA2-related modules.

To contextualize hippocampal LPC/PC dynamics at the cellular level, a Pearson correlation-based similarity analysis was performed using a previously published lipidomic dataset from astrocyte, neuron, and endothelial coculture systems exposed to glutamate-induced stress. This analysis compared post-ischemic hippocampal LPC/PC dynamics with LPC/PC responses in each coculture system.

LION/web was used for lipid ontology enrichment analysis of the full phospholipid species dataset. Ischemic samples were compared with time-matched sham controls to annotate lipid changes according to organelle-associated lipid categories and membrane biophysical properties, including membrane curvature, lateral diffusion, surface charge, and bilayer thickness. Thus, BioPAN, LipidSig, STRING, and LION/web were applied as complementary tools to infer phospholipid remodeling reactions, visualize species-level lipidomic patterns, prioritize candidate enzyme networks, and contextualize lipid changes at cellular, organellar, and membrane-property levels.

#### Tissue processing for histology, immunofluorescence, and *in situ*/tissue western immunoblotting

Perfusion-fixed brains from the second cohort were post-fixed in 4% paraformaldehyde for 48 h at 4°C, cryoprotected through sucrose gradients of 7%, 25%, and 30%, each for 48 h, and sectioned coronally at 50 μm using a cryostat. Sections were stored in 0.1 M PBS until processing for histological staining, immunofluorescence, or *in situ*/tissue Western immunoblotting.

#### Histology

For Nissl staining, sections were mounted on gelatin-coated slides, air-dried at room temperature, rehydrated through descending ethanol concentrations, washed with distilled water, incubated in 0.1% toluidine blue for 10 min, rinsed, dehydrated through ascending ethanol concentrations, cleared with xylene, and mounted with Consultmount. Sudan Black B and Oil Red O staining were used as qualitative histological approaches to visualize lipophilic structures. Sections were mounted on slides, dried for 24 h at 37°C, stained with either 0.3% Sudan Black B in 70% ethanol or 0.5% Oil Red O in 70% ethanol for 30 min at room temperature, washed with 70% ethanol, mounted with Fluoromount, and analyzed by bright-field microscopy. Images were acquired using bright-field microscopy for Nissl, Oil Red O, and Sudan Black B. Sudan Black B and Oil Red O panels were used as representative qualitative observations and were not used for quantitative comparisons.

#### Immunofluorescence and *in situ*/tissue western immunoblotting

Free-floating brain sections from perfusion-fixed animals of the second cohort were permeabilized with 0.3% Triton X-100 and blocked with 1% BSA in PBS. For conventional fluorescence microscopy, sections were incubated with anti-GFAP, mouse, 1:500, eBioscience 14-9892-82, followed by Alexa Fluor 594-conjugated secondary antibody, 1:1000. Neutral lipid-associated structures were labeled with BODIPY 493/503, 1:5000, Invitrogen D3922, and nuclei were counterstained with Hoechst, 1:1000. GFAP and BODIPY signals were quantified as integrated density within predefined regions of interest, as indicated in the figure legends.

For *in situ*/tissue Western immunoblotting, sections were incubated with antibodies against LPCAT1, rabbit, 1:500, Invitrogen PA5-106278; MBOAT1, rabbit, 1:500, MyBioSource MBS9236794; cPLA2, mouse, 1:500, Santa Cruz SC-454; phospho-cPLA2, rabbit, 1:500, Cell Signaling Technology 2831S; DGAT1, rabbit, 1:500, Invitrogen PA5-117074; and SREBF2, rabbit, 1:500, LifeSpan BioSciences LS-B1609. Infrared detection was performed using IRDye 680- and IRDye 800-conjugated secondary antibodies, LI-COR, 1:5000, according to the host species, and sections were scanned using the Odyssey imaging system. Negative controls incubated without primary antibodies did not show immunoreactivity. Images for fluorescence microscopy were captured using an Olympus IX81 inverted microscope equipped with an epifluorescence illumination system and appropriate filter sets. Quantitative *in situ*/tissue Western analyses were performed in predefined ipsilateral regions and were not designed to quantify hemispheric asymmetry.

#### Western blotting

Frontal cortex and ipsilateral hippocampus were dissected from non-perfused animals of the second cohort collected at 24 h, 1 month, and 4 months post-ischemia. Tissue samples were homogenized in ice-cold lysis buffer containing 150 mM NaCl, 20 mM Tris-HCl pH 7.4, 10% glycerol, 1 mM EDTA, 1% NP-40, and protease/phosphatase inhibitor cocktail, and centrifuged to obtain total protein extracts. Protein concentration was determined using the BCA assay.

Twenty micrograms of protein per sample were denatured in loading buffer containing 0.375 M Tris-HCl pH 6.8, 50% glycerol, 10% SDS, 0.5 M DTT, and 0.002% bromophenol blue, heated at 95°C for 3 min, and separated by 12% SDS-PAGE using a mini-PROTEAN system. Proteins were transferred to PVDF membranes at 250 mA for 2 h using a wet transfer system. Membranes were blocked with 5% non-fat dry milk in TTBS and incubated overnight at 4°C with primary antibodies against LPCAT1, MBOAT1, phospho-cPLA2, β-actin; and anti-HSP90, mouse monoclonal (AC88), Assay Designs SPA-830, lot 12010801. β-Actin was used as the loading control for LPCAT1 and MBOAT1, whereas HSP90 was used as the loading control for phospho-cPLA2. The following primary antibodies were used: anti-LPCAT1, rabbit, 1:500, Invitrogen PA5-106278; anti-MBOAT1, rabbit, 1:500, MyBioSource MBS9236794; anti-phospho-cPLA2, rabbit, 1:500, Cell Signaling Technology 2831S; and anti-β-actin, mouse, 1:1000, Invitrogen MA5-11869. After washing, membranes were incubated with species-specific secondary antibodies conjugated to IRDye 800CW or IRDye 680, LI-COR, 1:5000, for fluorescence detection. Although the antibody is reported to recognize phospho-cPLA2 at approximately 95 kDa, the predominant signal detected in rat brain extracts migrated at approximately 14 kDa and was therefore interpreted as an antibody-reactive band of unconfirmed molecular identity. Signals were visualized and quantified using the Odyssey Infrared Imaging System, Image Studio Software version 6.0. All samples were processed in parallel to reduce inter-assay variability.

#### Flow cytometry

Longitudinal tail-blood plasma samples collected before surgery at 0 h and during follow-up at 6 h, 24 h, 7 d, 15 d, 1 month, and 4 months from animals followed up to the 4-month endpoint were used for flow cytometry analysis, with final sample sizes indicated in the figure legends. Plasma samples were processed to remove cellular debris by centrifugation at 3000 rpm for 7 min at 4°C. For each sample, 100 μL of plasma was used for lipid staining. Neutral lipids were labeled with BODIPY 493/503 at a 1:5000 in PBS, and incubated at room temperature for 30–60 min protected from light. After incubation, samples were washed with PBS and centrifuged to remove excess dye.

Immunostaining was performed using primary antibodies against ApoA1, 1:500, Invitrogen 701239, and ApoB100, 1:500, Invitrogen MIA1609, diluted in PBS, followed by incubation at 4°C for 2 h. After three PBS washes, samples were incubated with Alexa Fluor 405-conjugated anti-rabbit, 1:2000, Thermo A-31556 and Alexa Fluor 647-conjugated anti-mouse, 1:2000, Thermo A-21235 secondary antibodies, or with directly conjugated primary antibodies, including ApoE-PE, 1:1000, BioLegend E6D7, for 30 min at 4°C in the dark. Final washes were performed with PBS, and samples were centrifuged prior to acquisition.

Flow cytometry was conducted using a CytoFLEX flow cytometer equipped with lasers capable of detecting BODIPY 493/503 and the corresponding fluorophores. BODIPY^+^ particle populations were gated according to size parameters and subdivided into P1, P2, and P3 populations. ApoE-, ApoA1-, and ApoB100-associated events were quantified within the BODIPY^+^ particle gate. Data were analyzed using FlowJo v10.

### Quantification and statistical analysis

Lipidomic statistical analyses were performed using the LipidSig web platform. For database preparation, lipid species with more than 80% missing data were removed. Remaining values were imputed based on the minimum value of each group. Group comparisons within LipidSig species-level analyses were conducted using t-tests for parametric data or Wilcoxon/Mann–Whitney tests for non-parametric data when comparing two groups. Multivariate analyses, including PCA, were also performed in LipidSig.

Quantitative analyses, including lipid ratios, neurological scores, longitudinal behavioral assessments, conventional Western blotting, immunofluorescence, *in situ*/tissue Western immunoblotting, and flow cytometry data, were performed using GraphPad Prism 8. Data normality was assessed using the Shapiro–Wilk test. Corrected infarct volume was analyzed using one-way ANOVA followed by Tukey’s multiple comparisons test. Homogeneity of variance for this analysis was evaluated using the Brown–Forsythe test.

Neurological scores were analyzed using ordinary two-way ANOVA with surgery and time as factors. Inclined plane performance was analyzed using two-way repeated-measures ANOVA with surgery and time as factors. Morris water maze acquisition was analyzed using ordinary two-way ANOVA at 1 month and two-way repeated-measures ANOVA at 4 months, with surgery and trial as factors. For these analyses, Šídák’s multiple comparisons test was used to compare sham and ischemic animals within each time point or trial, as applicable.

Plasma PC and LPC abundance, hippocampal and plasma LPC/PC ratios, and GFAP/BODIPY immunofluorescence data were analyzed using ordinary two-way ANOVA with surgery and time as factors, followed by Šídák’s multiple comparisons test comparing sham and ischemic animals within each time point. Selected hippocampal and plasma phospholipid-class comparisons were analyzed using unpaired two-tailed Student’s t-tests without correction for multiple comparisons. Longitudinal flow cytometry data were analyzed using one-way ANOVA followed by Tukey’s multiple comparisons test. Homogeneity of variance for these datasets was evaluated using Brown–Forsythe and Bartlett’s tests.

For conventional Western blot analyses, LPCAT1 and MBOAT1 band intensities were normalized to β-actin, whereas phospho-cPLA2 band intensities were normalized to HSP90. For each protein, brain region, and post-ischemic time point, individual values were expressed relative to the mean of the corresponding sham group, which was set to 100%. Statistical comparisons were then performed on the normalized individual values using multiple unpaired two-tailed Student’s t-tests, assuming a single pooled variance across comparisons within each post-ischemic time point. No correction for multiple comparisons was applied. Medial-level *in situ*/tissue Western immunoblotting data were analyzed using multiple unpaired two-tailed Student’s t-tests comparing sham and ischemic animals within each protein, brain region, and post-ischemic time point, without correction for multiple comparisons. Anterior-level *in situ*/tissue Western immunoblotting data and the cell-culture phospholipid analyses shown in Figures S3D–S3F was analyzed using multiple unpaired two-tailed Student’s t-tests. The two-stage step-up false discovery rate procedure of Benjamini, Krieger, and Yekutieli was additionally applied, with the desired false discovery rate set at Q = 1%. Figure significance labels were assigned according to the raw *p* values, whereas the corresponding FDR-adjusted q values are reported separately.

All quantitative data are presented as mean ± SD. Asterisks in figures indicate statistical significance as follows: ∗*p* < 0.05, ∗∗*p* < 0.01, ∗∗∗*p* < 0.001, and ∗∗*p* < 0.0001. For ANOVA-based analyses, figure significance labels reflect the corresponding post hoc adjusted *p* values. For multiple t-test analyses, significance labels reflect raw *p* values; when FDR correction was additionally applied, the corresponding adjusted q values are reported in Table S3. Detailed statistical information for each analysis, including the statistical test, biological sample size, test statistic, degrees of freedom, exact or raw *p* value, FDR-adjusted q value when applicable, and multiple-comparison procedure, is provided in Table S3.

## Supplemental information

Document S1. Figures S1–S6

Table S1. Phospholipid species analyzed in this study, related to Figures 2 and 3 Excel file listing the phospholipid species included in the hippocampal and plasma lipidomic datasets, including lipid class, structural category, functional category, total acyl chain length, total degree of unsaturation, and standardized nomenclature used for downstream bioinformatic analyses.

Table S2. Bioinformatically identified enzyme clusters associated with phospholipid remodeling, related to Figure 4 Excel file summarizing STRING-derived clustering of BioPAN-prioritized enzymes associated with phospholipid remodeling reactions. Enzymes are grouped into functional clusters based on protein–protein interaction networks, highlighting shared biological processes and pathways related to phospholipid metabolism and Land’s cycle-associated remodeling.

Table S3. Statistical reporting summary, related to Figures 1–7 and S1–S6 Excel file listing the statistical information for each quantified figure panel, including the statistical test used, biological sample size, test statistic, degrees of freedom, exact p values, and multiple-comparison correction when applicable.
