# Supplementary material for: Rat brain phospholipid remodeling correlates with plasma lipid clearance and late functional vulnerability after ischemic stroke
Source: iScience. 2026 Sep 5;29(9):117434. doi: 10.1016/j.isci.2026.117434 (PMC13594439; doi:10.1016/j.isci.2026.117434)
Supplement: Supplementary file 1 [file mmc1.pdf]

iScience, Volume *29*

## Supplemental information

# Rat brain phospholipid remodeling correlates with plasma lipid clearance and late functional vulnerability after ischemic stroke

**Iván Daniel Salomón-Cruz, Johanna Gutiérrez-Vargas, and Gloria Patricia Cardona-Gómez**

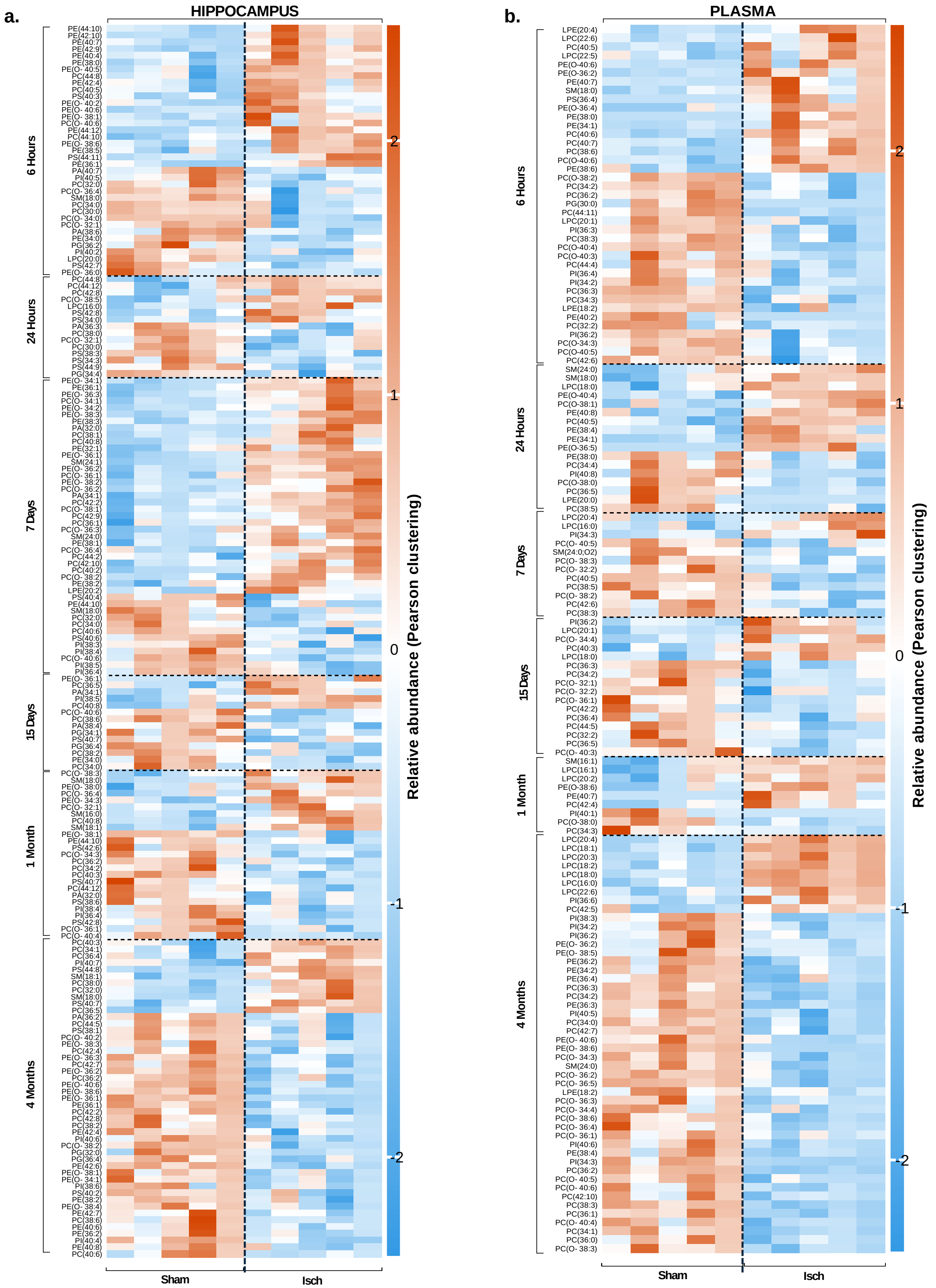


**Figure S1. Hierarchical clustering of phospholipid species after tMCAO generated by LipidSig. Related to Figure 2.** Heatmaps showing significantly altered phospholipid species in the hippocampus **(a)** and plasma **(b)** across post-ischemic time points. Lipid species abundance was expressed as molar percentage (%mol), and hierarchical clustering was performed using Pearson correlation as the similarity metric. Cell colors represent relative abundance patterns after Pearson correlation–based clustering, with red indicating higher relative values and blue indicating lower relative values within the clustered dataset. These heatmaps should not be interpreted as direct $log_2$ fold-change maps. Statistical comparisons between sham and ischemic animals were performed using Student's t-test for normally distributed data and Wilcoxon/Mann–Whitney test for non-normal data. Data are shown for n = 5 animals per group and time point.

| | Hippocampus | | | Plasma | | |
|---|---|---|---|---|---|---|
| | ***PCA Graph*** | ***Lipid Species*** | ***PCA1*** | ***PCA Graph*** | ***Lipid Species*** | ***PCA1*** |
| **6 Hours** | 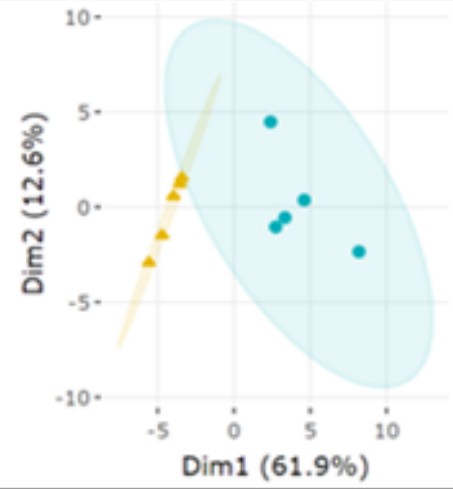 | PE(42:10) | 4.261 | 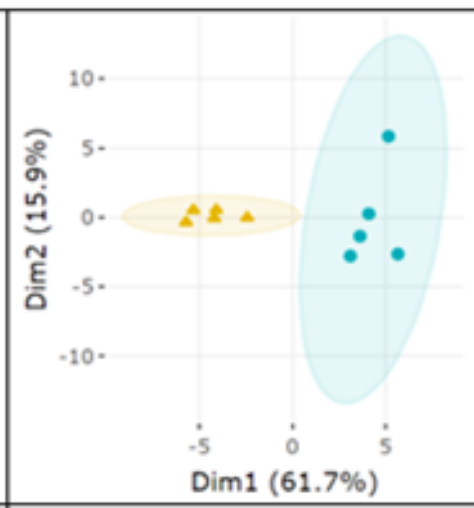 | PC(40:6) | 35.342 |
| | | PE(42:9) | 4.071 | | PC(36:3) | 33.197 |
| | | PE(44:10) | 3.847 | | PC(O- 40:4) | 31.461 |
| | | PE(38:0) | 3.803 | | | |
| | | SM(18:0) | 3.788 | | | |
| **24 Hours** | 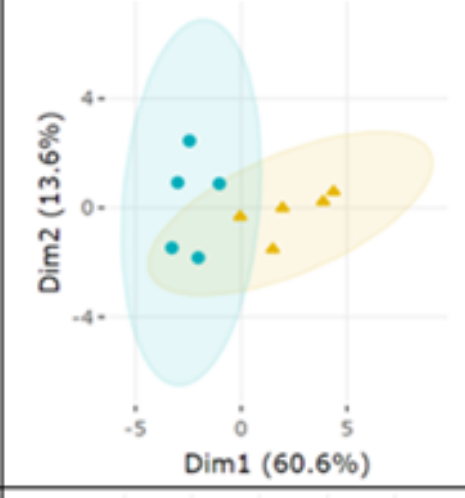 | PC(44:12) | 10.792 | 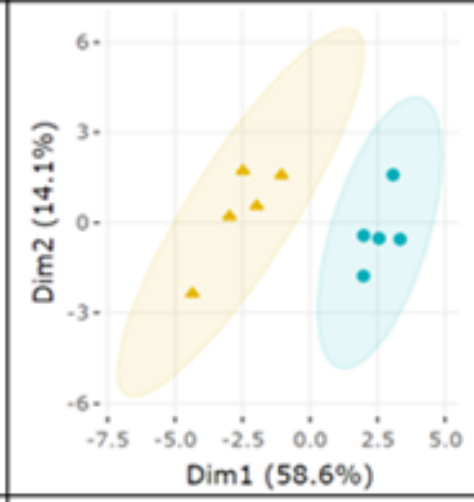 | PC(O- 38:0) | 10.323 |
| | | PC(44:8) | 9.557 | | PC(38:5) | 10.011 |
| | | PS(38:3) | 9.546 | | PE(O- 40:4) | 9.157 |
| | | PC(38:0) | 9.339 | | PC(36:5) | 9.057 |
| | | PC(30:0) | 9.309 | | SM(18:0) | 8.374 |
| **7 Days** | 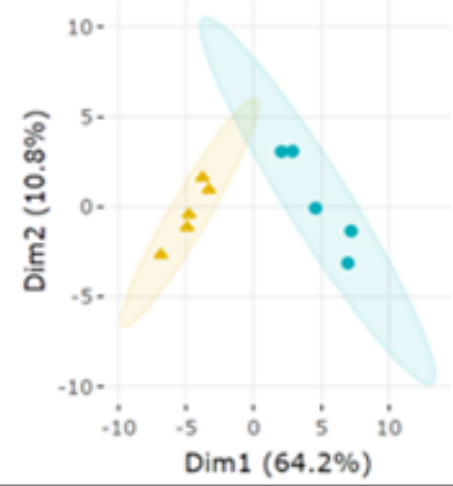 | SM(24:1) | 3.510 | 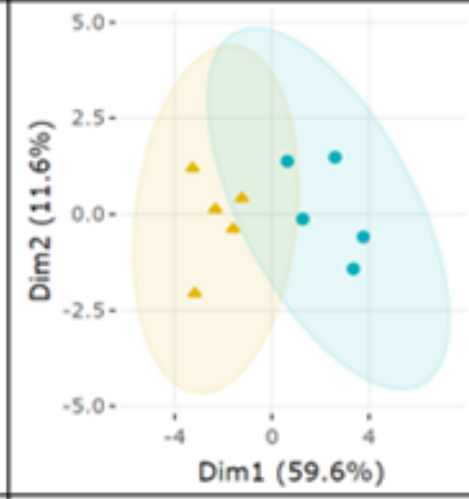 | PC(40:5) | 11.24969 |
| | | PE(O- 36:1) | 3.436 | | LPC(20:4) | 10.59059 |
| | | PC(42:2) | 3.265 | | LPC(16:0) | 10.00593 |
| | | PE(O- 36:3) | 3.177 | | PC(O- 32:2) | 9.72609 |
| | | PA(34:1) | 3.121 | | PC(O- 38:2) | 9.60818 |
| **15 Days** | 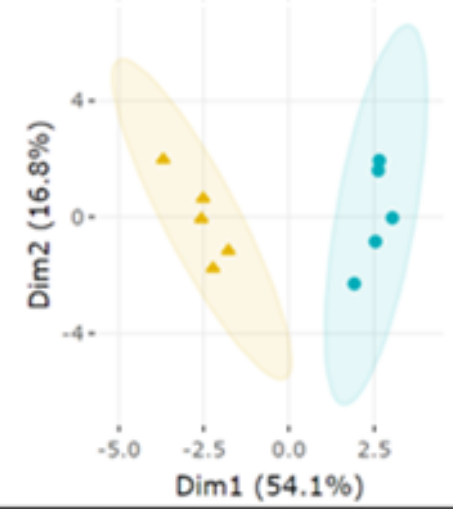 | PC(38:6) | 11.025 | 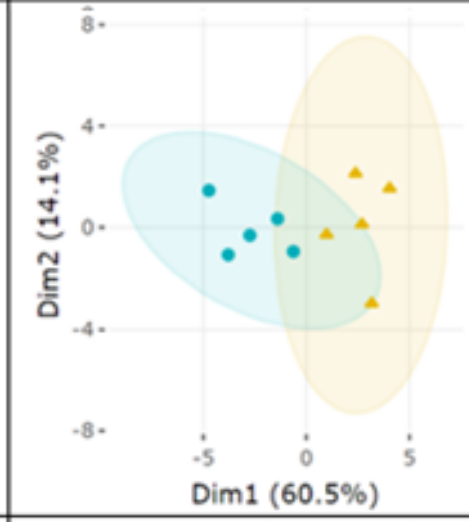 | PC(36:3) | 9.15417 |
| | | PC(40:8) | 9.804 | | LPC(18:0) | 8.61718 |
| | | PC(38:2) | 8.784 | | LPC(20:1) | 8.40357 |
| | | PC(O- 40:6) | 8.098 | | PC(34:2) | 7.4982 |
| | | PA(34:1) | 7.646 | | PC(O- 32:1) | 6.96551 |
| **1 Month** | 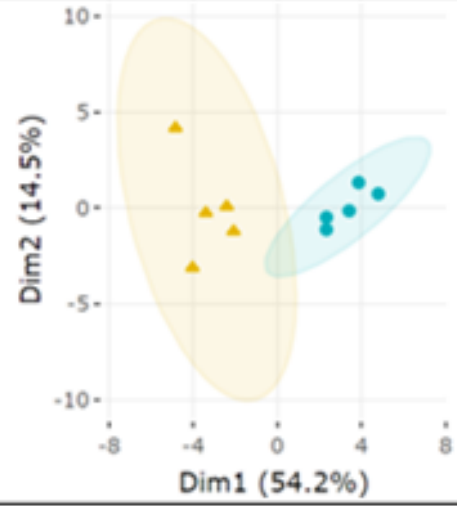 | PC(40:8) | 5.899 | 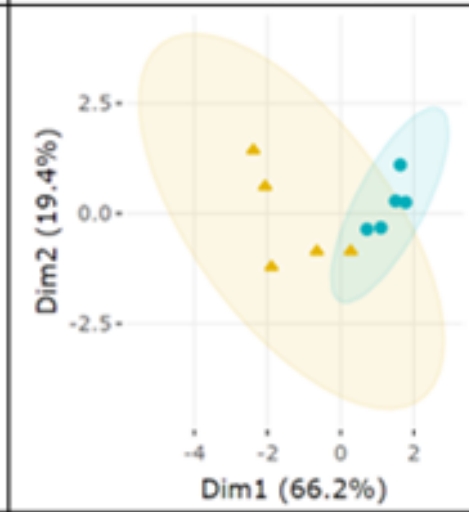 | SM(16:1) | 29.94001 |
| | | PC(40:3) | 5.486 | | PC(O- 38:0) | 29.24786 |
| | | SM(18:1) | 5.369 | | PC(34:3) | 25.46091 |
| | | SM(18:0) | 5.300 | | PE(O- 40:4) | 15.35122 |
| | | SM(16:0) | 5.244 | | | |
| **4 Months** | 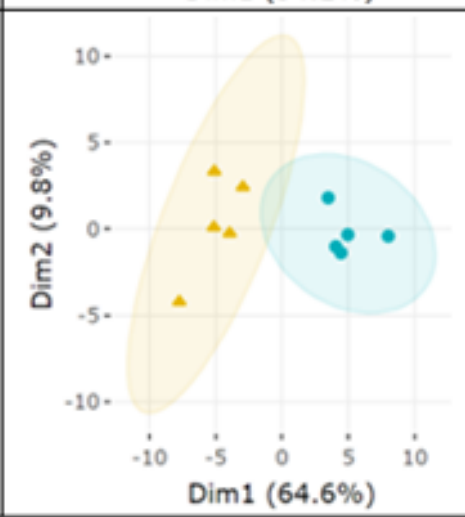 | PE(O- 40:6) | 3.142 | 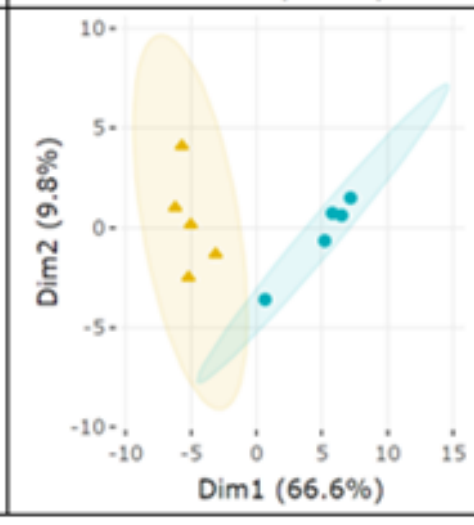 | PC(36:2) | 3.01611 |
| | | PC(42:2) | 2.877 | | LPC(18:1) | 2.87176 |
| | | PC(32:0) | 2.697 | | PC(38:3) | 2.79621 |
| | | PE(O- 38:6) | 2.697 | | PC(34:2) | 2.76845 |
| | | PC(34:1) | 2.6739 | | PI(34:3) | 2.7628 |

Sham Ischemia

**Figure S2. PCA-based identification of discriminant phospholipid species after tMCAO generated by LipidSig. Related to Figure 2.** Principal component analysis (PCA) showing the top five phospholipid species contributing to group separation between sham and ischemic animals across post-ischemic time points in the hippocampus and plasma. Lipid species abundance was expressed as molar percentage (%mol). The x- and y-axes represent the principal components explaining the largest proportion of variance in the dataset. The highlighted lipid species correspond to those with the highest contribution or loadings to PCA-based separation at each time point. Data are shown for n = 5 animals per group and time point.

a.

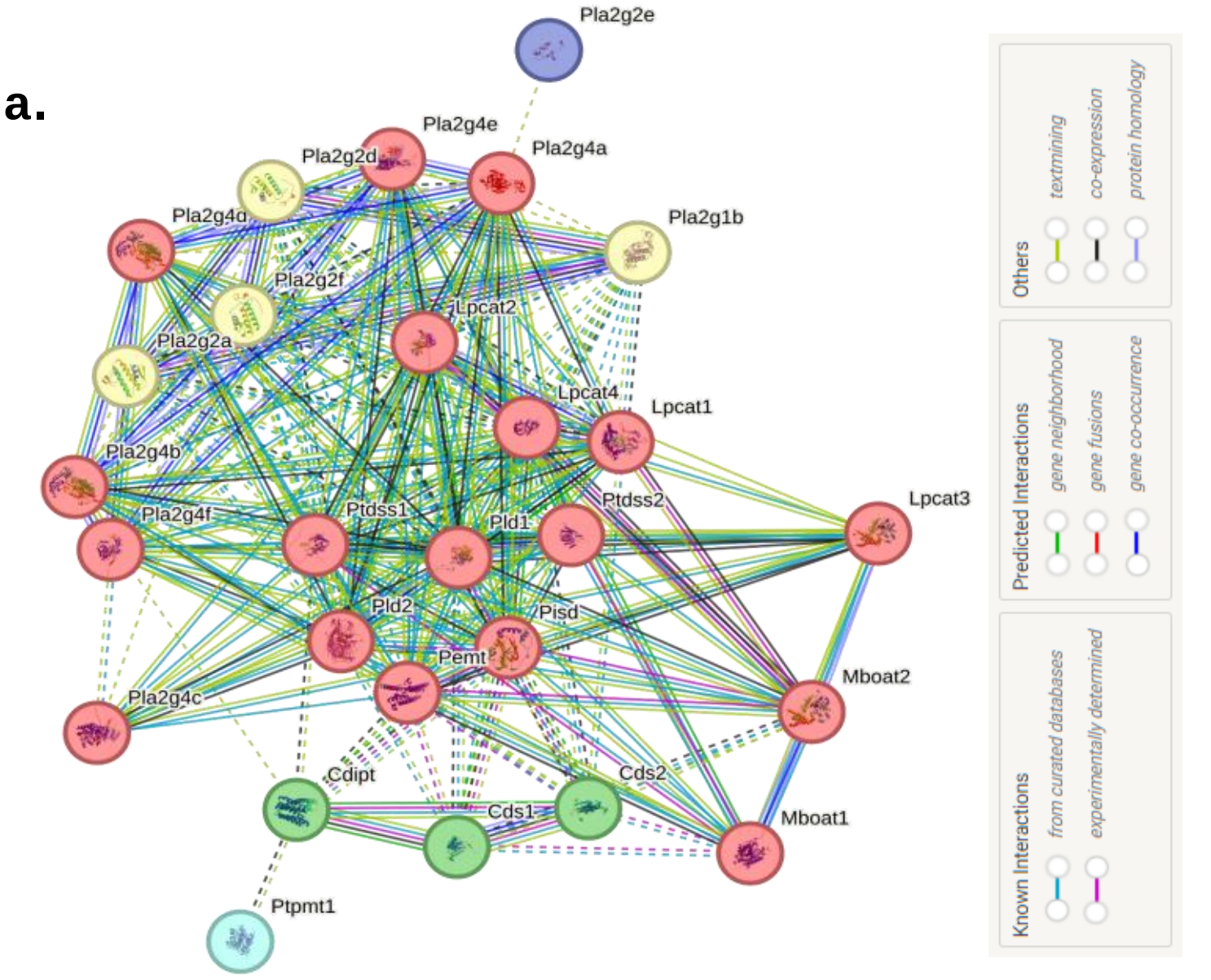


b.

| bubble | cluster Id | gene count | protein names |
|---|---|---|---|
| | Cluster 1 | 18 | Lpcat1, Lpcat2, Lpcat3, Lpcat4, Mboat1, Mboat2, Pemt, Pisd, Pla2g4a, Pla2g4b, Pla2g4c, Pla2g4d, Pla2g4e, Pla2g4f, Pld1, Pld2, Ptdss1, Ptdss2 |
| | Cluster 2 | 4 | Pla2g1b, Pla2g2a, Pla2g2d, Pla2g2f |
| | Cluster 3 | 3 | Cdipt, Cds1, Cds2 |
| | Cluster 4 | 1 | Ptpmt1 |
| | Cluster 5 | 1 | Pla2g2e |

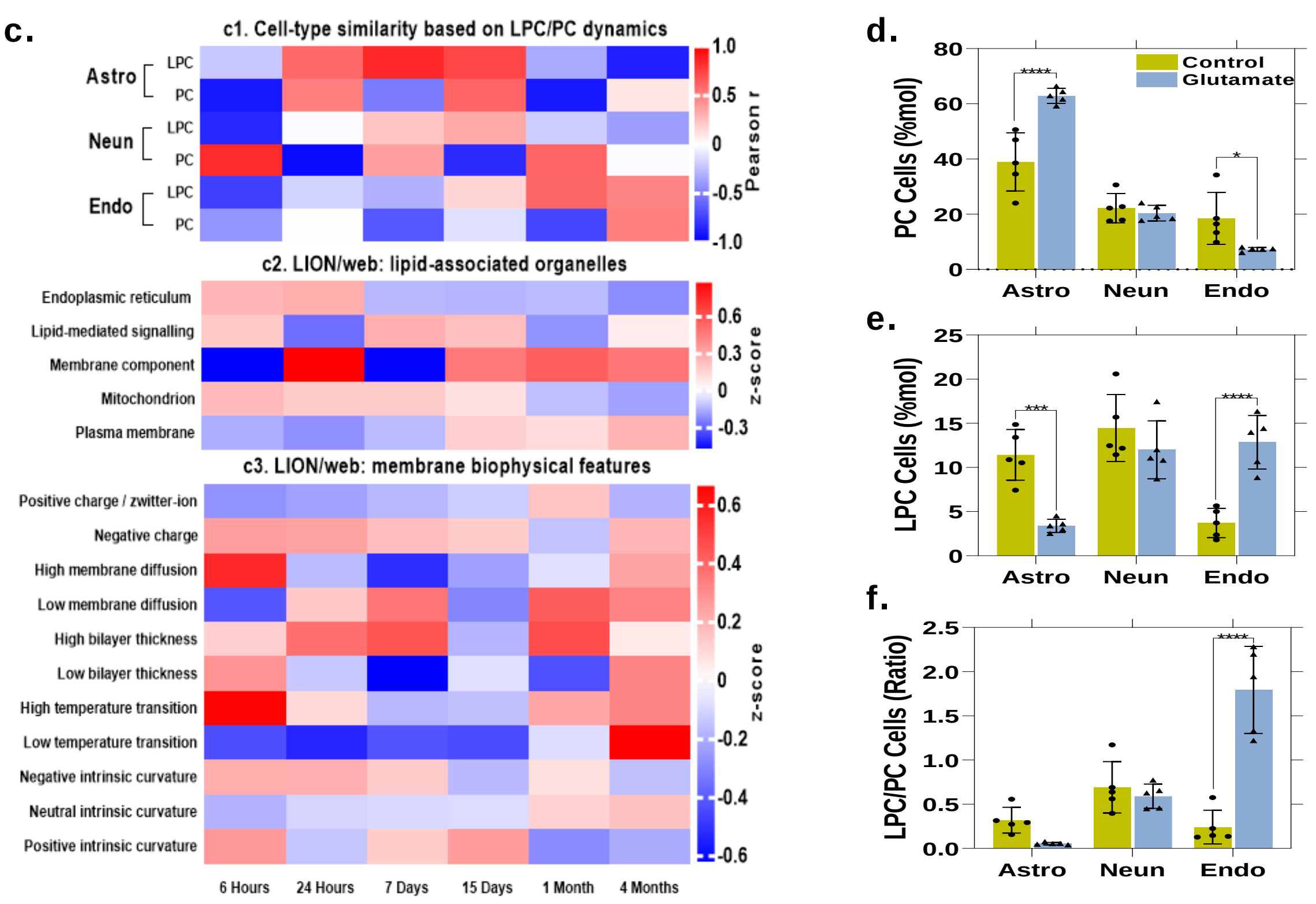


**Figure S3. Bioinformatic prioritization of phospholipid remodeling pathways and cell-type/membrane-associated lipid signatures. Related to Figures 3 and 4. (a)** STRING protein interaction network of BioPAN-derived enzyme candidates associated with phospholipid remodeling reactions. Nodes represent lipid-regulatory enzymes, and edges represent evidence-based protein–protein functional associations. **(b)** Summary of STRING-derived clusters highlighting groups of enzymes with related phospholipid remodeling functions. **(c)** Bioinformatic heatmap analyses contextualizing post-ischemic phospholipid remodeling. The first section shows a Pearson correlation–based similarity analysis comparing hippocampal LPC/PC dynamics after tMCAO with LPC/PC responses from astrocyte, neuron, and endothelial coculture lipidomic datasets. The second and third sections were generated using LION/web from the full phospholipid species dataset comparing ischemic animals with time-matched sham controls. These sections summarize lipid ontology enrichment related to organelle-associated lipid categories and membrane biophysical properties, respectively. **(d–f)** Bar graphs showing PC abundance, LPC abundance, and the LPC/PC ratio in astrocyte, neuron, and endothelial coculture datasets. For panels **(d–f)**, control and glutamate-treated groups were compared using unpaired two-tailed Student's t-tests, followed by false discovery rate (FDR) correction across cell-type comparisons using the two-stage step-up method of Benjamini, Krieger, and Yekutieli, with the desired FDR set at Q = 1%. The corresponding FDR-adjusted q values are reported in Table S3. Asterisks indicate significance based on raw p values. Significant changes are indicated as *$p < 0.05$, **$p < 0.01$, and ***$p < 0.001$. Data are shown for n = 5 per group. Quantitative data are presented as mean ± SD.

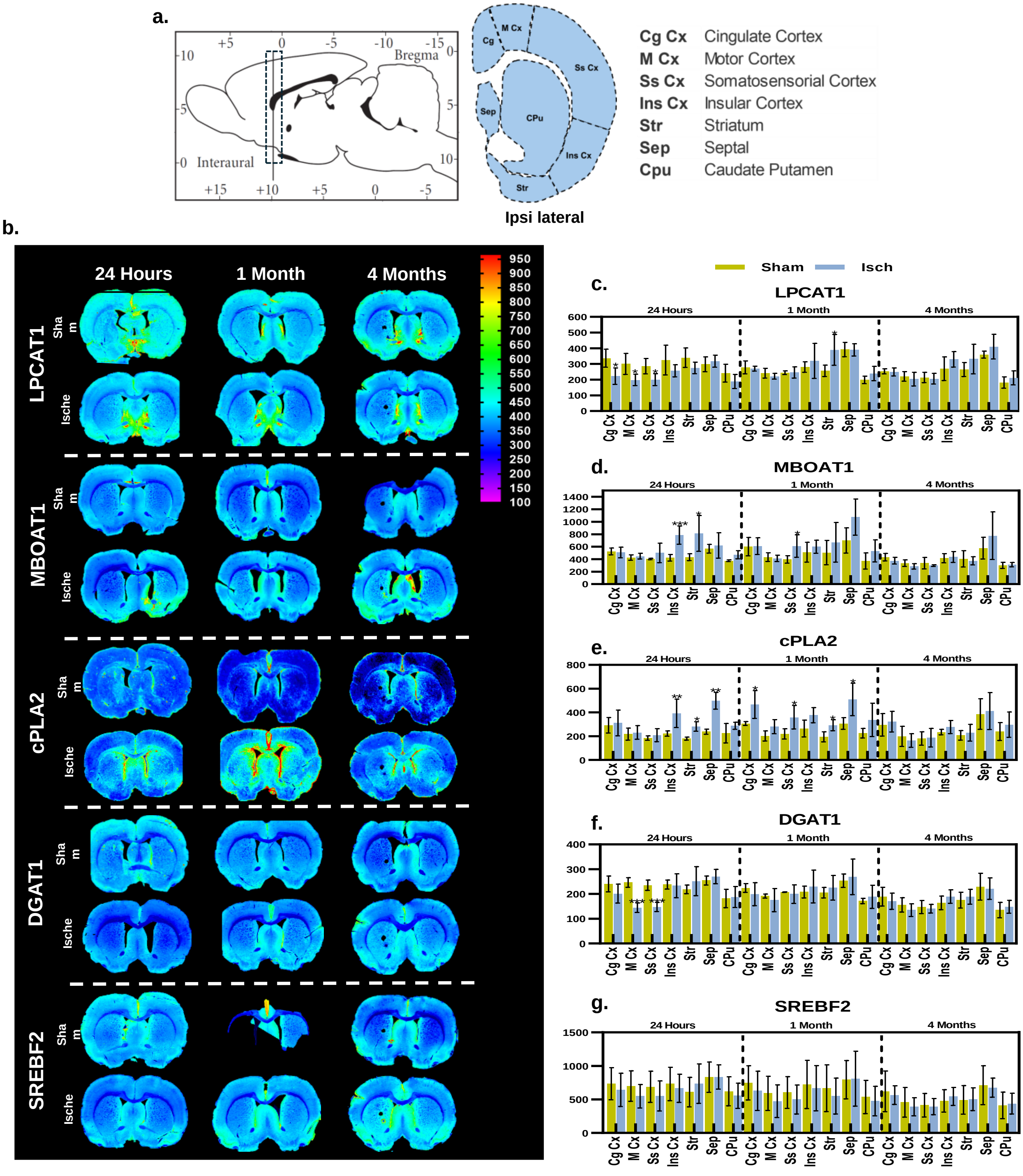


**Figure S4. Regional protein levels of Land's cycle and lipid storage regulators at anterior bregma levels after tMCAO. Related to Figure 5. (a)** Schematic representation of the anterior bregma level and analyzed ipsilateral brain regions. **(b)** Representative in situ/tissue Western immunoblotting images showing protein signal distribution across post-ischemic time points. **(c–g)** Regional quantification of protein levels at 24 h, 1 month, and 4 months post-ischemia. Statistical analysis was performed using multiple unpaired two-tailed Student's t-tests comparing sham and ischemic animals within each brain region and time point. The two-stage step-up false discovery rate procedure of Benjamini, Krieger, and Yekutieli was additionally applied, with the desired false discovery rate set at Q = 1%. Asterisks indicate significance based on raw p values, whereas the corresponding FDR-adjusted q values are reported in Table S3. Significant changes are indicated as *p < 0.05, **p < 0.01, and ***p < 0.001. Data are shown for n = 3 animals per group and time point. Quantitative data are presented as mean ± SD.

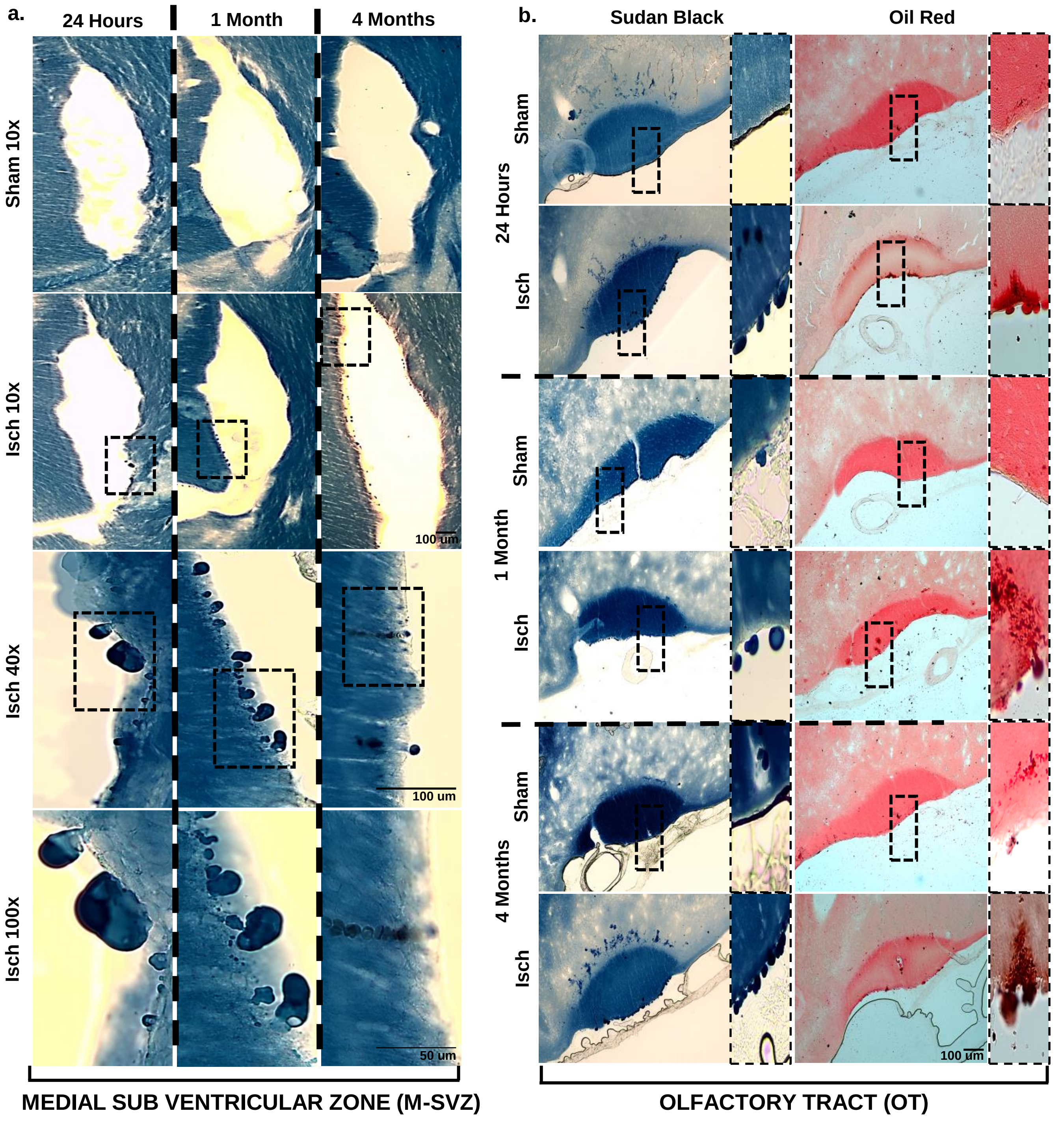


**Figure S5. Representative qualitative histology of lipophilic structures in periventricular and olfactory-associated regions after tMCAO. Related to Figure 6. (a)** Representative qualitative Sudan Black images showing spherical lipophilic structures along the lateral ventricle–associated anterior SVZ/periventricular region at 24 h, 1 month, and 4 months post-ischemia. Scale bars: 100 µm for the 10× and 40× images, and 50 µm for the 100× image. **(b)** Representative qualitative Sudan Black and Oil Red O images showing lipophilic structures in the olfactory tract at 24 h, 1 month, and 4 months post-ischemia. Scale bars: 100 µm. These panels are presented as qualitative histological observations to illustrate the morphology and regional distribution of lipophilic material and were not used for quantitative comparisons. Representative images were selected from n = 3 animals per group and time point.

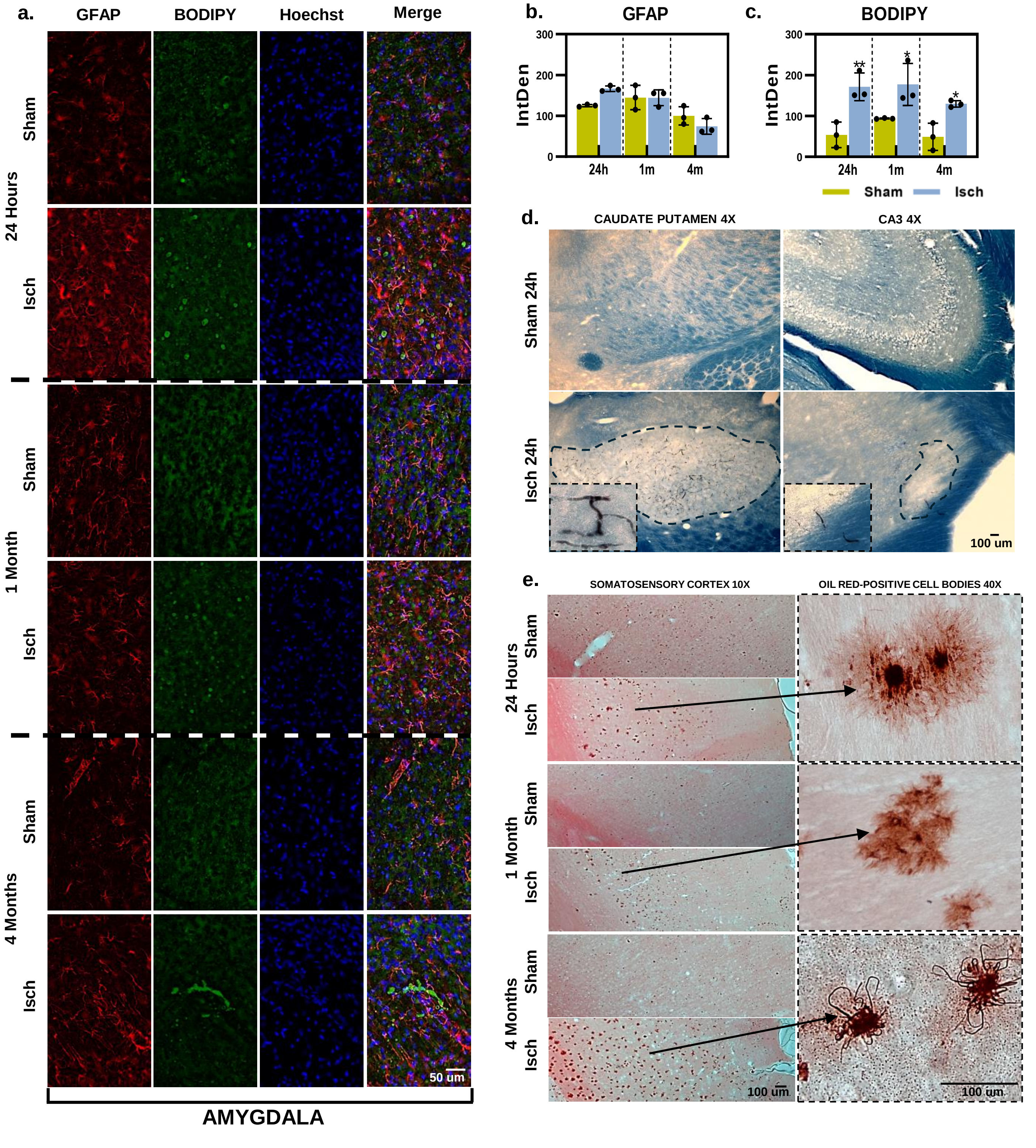


**Figure S6. Region-specific lipophilic structures and astroglial reactivity in the post-ischemic amygdala. Related to Figure 6. (a)** Representative immunofluorescence images of GFAP and BODIPY 493/503 in the amygdala across post-ischemic time points. Scale bars: 50 µm. (b) Quantification of GFAP immunofluorescence signal. **(c)** Quantification of BODIPY 493/503 signal. **(d)** Representative qualitative Sudan Black staining at 24 h post-ischemia. Scale bars: 100 µm. **(e)** Representative qualitative Oil Red O staining showing lipid-rich cell-like structures during post-ischemic progression. Scale bars: 100 µm. GFAP and BODIPY signals were quantified as integrated density within predefined regions of interest. Sudan Black and Oil Red O panels are shown as representative qualitative histological images and were not used for quantitative comparisons. Statistical analysis for GFAP and BODIPY quantification was performed using ordinary two-way ANOVA, with Šídák's multiple comparisons test used for sham versus ischemic comparisons within each time point when applicable. Significant changes are indicated as *p < 0.05, **p < 0.01, ***p < 0.001, and ****p < 0.0001. Data are shown for n = 3 animals per group and time point. Quantitative data are presented as mean ± SD.
